# A primer on expert elicitation with IDEA (Investigate Discuss Estimate Aggregate) for rare disease trialists

**DOI:** 10.1007/s44162-026-00253-8

**Published:** 2026-11-04

**Authors:** Arlene Jiang, Danielle A. Baribeau, John J. Mitchell, Bernard Thébaud, Nicola A. M. Wright, Anna Heath

**Affiliations:** 1 Department of Psychiatry, University of Toronto, Toronto, ON Canada; 2 Department of Psychiatry, Hospital for Sick Children, Toronto, ON Canada; 3 Autism Research Centre, Holland Bloorview Kids Rehabilitation Hospital, Toronto, ON Canada; 4 Montreal Children’s Hospital, McGill University Health Centre, Montreal, QC Canada; 5 Regenerative Medicine Program, The Ottawa Hospital Research Institute, Ottawa, ON Canada; 6 Department of Cellular and Molecular Medicine, University of Ottawa, Ottawa, ON Canada; 7 Department of Pediatrics, Cumming School of Medicine, University of Calgary, Calgary, AB Canada; 8 Child Health Evaluative Sciences, The Hospital for Sick Children, Toronto, ON Canada; 9 Division of Biostatistics, Dalla Lana School of Public Health, University of Toronto, Toronto, ON Canada; 10 Department of Statistical Science, University College London, London, UK

**Keywords:** Expert elicitation, Rare disease, Clinical trial, Investigate Discuss Estimate Aggregate (IDEA), Bayesian

## Abstract

In clinical trials for rare diseases, small patient populations constrain recruitment, limiting data collection. Expert elicitation, the process of extracting expert beliefs into quantitative expressions, can act as a source of evidence in a setting with limited data. Expert judgement can be combined with trial data through the prior distribution in a Bayesian trial. This approach makes the most use out of the available information and possible trial recruitment, unlike conventional approaches that rely on trial data alone. Previous elicitations for rare disease trial prior distributions have used ad hoc approaches, rather than an established protocol designed to limit biases in judgement. This practical guide introduces rare disease clinical investigators to expert elicitation using the IDEA (Investigate, Discuss, Estimate, Aggregate) protocol. IDEA is a structured elicitation technique where experts individually estimate quantities, discuss their answers, then revise their estimates before aggregation into a final distribution. In an elicitation for a trial, experts can either estimate the control arm response or the treatment effect. The elicitation estimates then inform the design and analysis of the trial. IDEA has previously been described in a general context, but this paper provides tailored recommendations for conducting an elicitation for a clinical trial for a rare disease. By outlining practical steps to using IDEA, we aim to promote more structured, transparent practices that will increase the uptake and acceptance of expert elicitation to improve clinical trials for rare diseases.

**Supplementary Information:**

The online version contains supplementary material available at https://doi.org/10.1007/s44162-026-00253-8.

## Background

Rare diseases have a prevalence of fewer than 1 person in 1500 or fewer than 1 person in 2000 (a definition that varies regionally) [1–3]. Despite their low individual prevalence, there are over 7000 rare diseases, which collectively pose a disease burden on 300 million people worldwide [4]. This burden is exacerbated by the limited treatment options for rare diseases, which when available are often overly costly. Clinical trials that establish the safety and efficacy of new therapies are urgently needed for rare diseases. However, the low prevalence of rare diseases creates design and recruitment challenges for clinical trials. Innovative trial designs are necessary in order to develop effective and affordable therapies for rare diseases.

Bayesian methods provide an alternative framework that is well-suited to the rare disease trial setting [5]. While conventional frequentist methods draw conclusions from trial data alone, Bayesian statistics incorporate all available information into the analysis [6–9]. Information external to the trial is embedded into a prior distribution, which reflects beliefs about the treatment effect on the primary outcome before the trial is conducted. A Bayesian analysis combines trial data with the prior distribution to generate a posterior distribution, representing updated beliefs about the treatment effect [7]. The prior distribution can either contain information about the difference in the primary outcome between intervention arms (i.e. treatment effect) or a single arm’s response. For instance, if responses are only known under standard-of-care, which tends to be more familiar to clinicians, a prior distribution for could be constructed for the response in that arm only. Conversely, if clinicians have experience with both the comparator and the intervention, then estimates for both arms could be obtained. If there is no clinical experience with an intervention (i.e. a novel treatment), a non-informative prior should be implemented. Prior distributions should make use of all available information during trial design [7]. While prior distributions are ideally constructed with empirical evidence from past studies [10, 11], expert knowledge can also be used in a prior distribution [12, 13].

In rare diseases, expert knowledge from past clinical experiences is frequently used to inform medical decisions. As there are limited empirical data on rare diseases, consulting with networks of experts provides critical insights for medical practice [14, 15]. Similarly, expert knowledge can be formally quantified as a prior distribution through structured elicitation processes to inform the design of clinical trials for rare diseases. Prior elicitation is best suited to limited data settings when there is expertise in caring for the relevant patient population.

Expert elicitation is the process of quantifying and combining expert knowledge. It is often used to generate evidence to support decision-making in settings with limited data. During an elicitation, experts are asked to quantify their uncertainty about an unknown parameter. In the context of a clinical trial elicitation, this unknown parameter is typically either the treatment effect or the control arm response. A facilitator converts each expert’s estimates into a distribution representing an expert’s belief about the parameter. Individual expert judgements are then combined mathematically and/or through group discussion. This effectively transforms multiple experts’ subjective beliefs into a statistical distribution, which can be used as a prior distribution. The aggregation process is further described in the Post-Workshop section.

Expert elicitation methodology has been developed for a breadth of applications. Bojke et al. (2021) performed a review of existing elicitation guidelines, in their presentation of the Medical Research Council reference protocol designed for elicitation for health technology assessment [16]. Their review identified 16 unique protocols, of which 11 were created for a specific domain (i.e. ecology, food safety, environmental protection, insurance, risk and reliability, nuclear, environmental health). This finding highlights the need for expert elicitation across a range of fields, as well as the need to tailor protocols for field-specific considerations. In particular, there is growing interest and methods development in elicitation for Bayesian trials [13, 17–19].

Table 1 presents rare disease trials that used elicitation from a review by Azzolina (2021), alongside an informal updated search performed on June 1, 2025 [17]. The use of elicitation and Bayesian methodology enables the completion of trials with small sample sizes, by maximizing the information available with a small number of patients. By a frequentist sample size calculation, which is designed to provide a binary yes or no answer to the research question, the trial by Hampson (2015) required 513 patients for 90% power [20]. However, their team expected to recruit only 40 patients. Under a frequentist framework, conducting this study would waste research resources as the trial would be underpowered to detect an effect. In contrast, Bayesian methods allow investigators to estimate the probability of treatment benefit on a continuum rather than relying on a single threshold for significance. Hampson et al. (2015) derived a prior distribution through expert elicitation in order to integrate existing knowledge into their Bayesian analysis. This highlights the value in using elicitation and Bayesian methods in the rare disease setting.

The previous rare disease trials presented in Table 1 employed ad hoc methods, rather than standardized elicitation protocols. Only one study, Ramanan (2019), used an existing elicitation methodology [20], which was created for a previous rare disease trial. The use of ad hoc approaches demonstrates the need for practical recommendations for how to employ a standardized protocol in rare disease trials. Standardized protocols are designed to limit biases common with expert judgement, improving the quality of the elicitation [21]. We believe that the IDEA protocol is the structured elicitation method that best fits rare disease trials. The IDEA protocol is a simple, flexible approach that can be delivered remotely or face-to-face, which suits the rare disease context where experts tend to be geographically separated. This paper is also informed by our practical experience with using the IDEA protocol to elicit prior distributions for two clinical trials [22, 23].

**Table 1:** Examples of expert elicitation in rare disease trials

Trial	Disease	Age Group	Outcome (Outcome Type)	Elicitation Framework Used
Moatti (2013) [24]	Stage II or III multiple myeloma	Adult	Overall (Survival)	None
Hampson (2015) [20]	Polyarteritis nodosa	Pediatric	Disease remission within 6-months (Binary)	None
Thall (2017) [25]	Steroid-dependent idiopathic nephrotic syndrome	Pediatric	No occurrence of relapse during the 24 months of follow-up (Binary)	None
Ramanan (2019) [26]	Chronic nonbacterial osteomyelitis (CNO)/chronic recurrent multi-focal osteomyelitis (CRMO)	Pediatric	Change in pain score from baseline at 26 weeks with Visual Analog Scale (Continuous)	Hampson (2015) [20]
Papadopoulou (2024) [27]	Juvenile dermatomyositis (JDM)	Pediatric	Percentage achieving clinically inactive disease, and off glucocorticoids at 12 months (Binary)	None

## Developing an elicitation

**Fig. 1:**
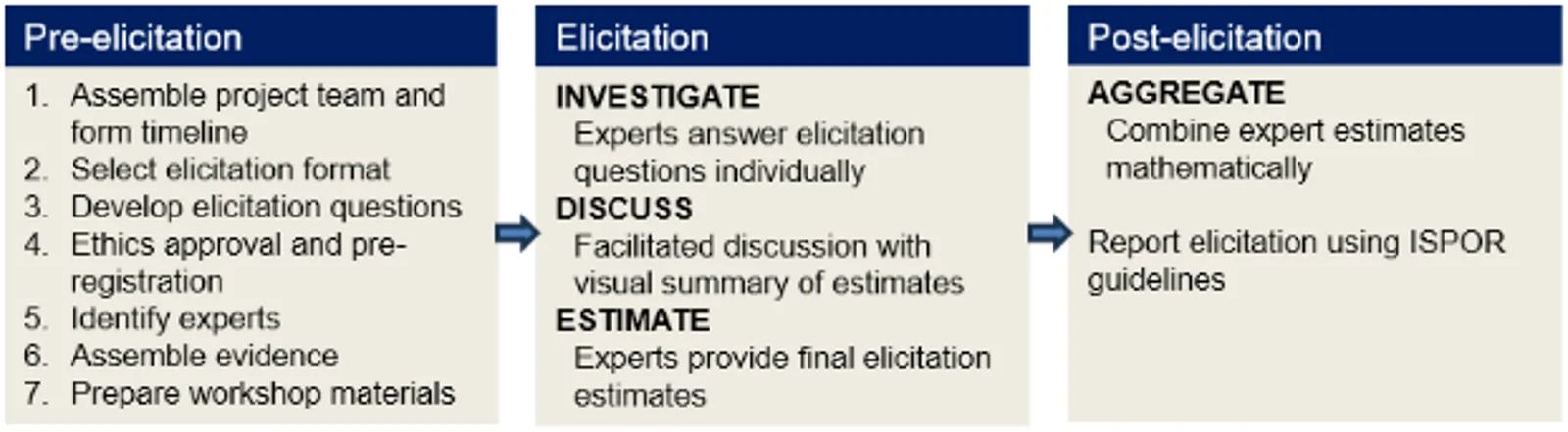
Flowchart of expert elicitation process for rare disease trialists based on IDEA

The following guide provides practical steps for developing an elicitation to construct a prior distribution for a clinical trial in a rare disease. Depending on whether your experts have experience with both the intervention and the comparator or the comparator alone, this prior distribution will either describe existing knowledge on the treatment effect or the control arm response. The IDEA elicitation generally involves four phases: (1) Investigate, (2) Discuss, (3) Estimate, (4) Aggregate. During an elicitation, experts first individually investigate the elicitation questions, discuss their answers as a group, then individually estimate the elicitation quantities. Finally, an aggregate is calculated, combining expert estimates. This guide explains how to prepare and carry out an elicitation, summarized in Fig. 1.

### Preparation

Before developing your elicitation, the trial question and its PICO (population, intervention, comparator, outcome) should be clearly defined [28]. Being clear on your research question will help ensure that your elicitation is relevant to your trial.

#### Step 1. Assemble your project team and establish your timeline

The IDEA protocol lists four roles that are required for an elicitation: (1) problem owner, (2) coordinator, (3) facilitator, (4) analyst. The problem owner is the trial’s principal investigator and is responsible for identifying quantities to elicit and recruiting experts. The coordinator oversees the elicitation and should be familiar with your institution’s research ethics board process. The facilitator manages the elicitation session. They should be a clear communicator, have a background in statistics, and be aware of biases with expert judgement (the cited works provide background on these biases) [29–33]. The analyst processes and aggregates the elicited responses. These four roles can be split between the trial’s principal investigator and research coordinator. However, an external team could be recruited to support with the elicitation process, if it is unfamiliar. Your team should then collectively decide on a project timeline. The preparation stage is estimated to require 2 weeks to 4 months, while the elicitation is estimated to require 2 to 6 weeks (depending on the mode of elicitation) [34]. You should develop a timeline based on these guidelines as well as your elicitation context.

#### Step 2. Select your elicitation format

One important consideration for your elicitation is its mode of delivery. In choosing your elicitation format, consider your experts’ geographic locations, their availability, as well as your budget. With the low prevalence of rare diseases, experts tend to be geographically dispersed. Furthermore, you may choose to recruit practicing clinicians, who have multiple competing demands on their schedules.

You might opt to deliver your elicitation as an in-person workshop. This format typically leads to high engagement as well as acceptance of the outcomes elicited [32, 35]. Past research has also shown that an in-person elicitation workshop tends to take less time than other formats [36]. However, an in-person session would require significant time commitments from the experts as well as the budget for flights and hotel rooms. This may be overly time-consuming for the experts and infeasibly expensive for your team. Previous in-person elicitations for clinical trials have recruited experts present at society meetings, an approach that could reduce the time commitment and costs for your elicitation. However, your rare disease may not have a dedicated society meeting, Furthermore, the experts that attend the society meeting may not be representative of your overall population of eligible experts (see step 5).

The IDEA protocol can flexibly accommodate remote, asynchronous elicitation. In this format, experts answer elicitation questions and review each other’s answers over email. This is a convenient, economical approach that would work around the busy schedules of your experts, allowing them to answer the elicitation questions on their own time. A downside to this flexibility is that experts tend to be less engaged in this remote elicitation format [32].

An alternative approach is to conduct your elicitation in real-time with videoconferencing software (i.e. Zoom, Microsoft Teams). Virtual, real-time elicitation allows experts to actively engage in discussion, while operating with a lower budget and time commitment requirement than an in-person session. We previously adopted this format for two clinical trial elicitations, during which we observed high levels of engagement from our experts. Furthermore, this format allowed us to accommodate the busy schedules of our experts, who could attend one of three planned elicitation workshops. Given the flexibility, feasibility, and engagement with this approach, we encourage the use of the virtual, real-time method in future elicitations.

#### Step 3. Develop your elicitation questions

Your elicitation will quantify beliefs about patient responses as measured by your trial’s primary outcome. Defining your primary outcome is crucial to both your trial and elicitation. However, choosing an outcome can be challenging given the limited data on the natural history of rare diseases [37]. Furthermore, rare diseases are often heterogenous, meaning that patients often experience different symptoms. Therefore, a single outcome may not adequately capture the patient experience of a rare disease. To address these challenges, rare disease patients and families with lived experience in disease course and their most relevant symptoms should co-develop your primary outcome [38, 39]. Above all, your outcome should be meaningful to patients, capturing how individuals feel, function, or survive [40–42]. The process of selecting a primary outcome, and an associated measurement instrument, is beyond the scope of this papers; readers are directed other resources (e.g., the Food and Drug Administration Guidance Documents for Rare Disease Drug Development) [43–45].

Another consideration in the rare disease setting is choosing an outcome that will minimize trial sample size requirements [46]. In the interest of statistical efficiency, continuous and repeated measure outcomes tend to provide greater power [47, 48]. Composite endpoints can also address disease heterogeneity and increase power [48].

Once you arrive at a relevant primary outcome, you should decide whether to elicit the response in the comparator arm alone or in both comparator and intervention arms. This choice will depend on whether your experts have experience with treating patients with the intervention. If the intervention is novel and unfamiliar, we suggest eliciting for just the comparator arm. If instead the intervention is used in practice, both arms can be elicited.

Next, you can begin to construct your elicitation questions. The IDEA protocol states that questions should have a format that is familiar to the experts. As such, we suggest framing questions on everyday clinical quantities. For example, you could ask, “Out of 20 patients you see with this rare disease treated with standard-of-care, how many typically experience the primary outcome?”. Similarly, for continuous (score) outcomes, you may have experts consider, “Among 20 patients you see with this rare disease treated with standard-of-care, what is the average score you would expect to see in the primary outcome?”. Previous elicitation exercises for more common diseases have used a similar framing, while asking for responses among 100 similar patients [22, 23]. In the supplement (Supplementary Material 1), we provide specific examples of questions drafted by rare disease clinical investigators. These examples are also accompanied by a clinical scenario that describes a typical patient that would be recruited to the hypothetical trial. We recommend drafting a similar scenario based off the inclusion and exclusion criteria for your trial.

Quantities can be elicited with a four-step elicitation method [49], by asking the following four questions.

1. What is the lowest plausible value?
2. What is the highest plausible value?
3. What is your best estimate?
4. What confidence do you have that the value lies between your lowest and highest plausible values? Pick a value between 50 and 100%.

In these questions, the lowest and highest plausible values represent the limits of what an expert considers reasonably possible. The best estimate is what the expert would believe to happen on average. The confidence level quantifies how sure an expert is that their lowest and highest plausible values contain the truth.

To help familiarize experts with the four-step elicitation process, you should draft a practice question to provide to the experts ahead of your elicitation questions. This question can be unrelated to the subject of your elicitation. You might ask, “At 12pm tomorrow, what will the temperature be in Celsius?”, with the four steps described above. Including a practice question will help orient the experts around the process of elicitation before the actual elicitation.

The elicitation questions should also be reviewed by two independent subject matter experts. The reviewers should ensure that the questions are clear, interpretable, and within the domain of expertise of the workshop participants. The review process helps minimize ambiguity and ensures that the elicitation is framed in clinically meaningful and relevant terms.

To elicit a treatment effect, we recommend having experts separately estimate their beliefs about the primary outcome response in the comparator and intervention arm. These quantities more directly translate to a physician’s clinical experience, compared to more abstract measures of effect (i.e. odds ratio/hazard ratio). However, experts estimating both quantities are likely to anchor one estimate on the other, which means that we should not consider these to be independent quantities. Thall et al. developed a method of constructing a bivariate prior distribution across the beliefs around the probability of a binary outcome under intervention and comparator, incorporating covariates provided by their experts [25]. They asked experts about their years of experience in medicine, their years of experience in the disease area, the number of patients treated for the disease, the number of patients they had prescribed the comparator, the number of patients they had prescribed the intervention, whether they received training in clinical trials, and whether they received training in statistics. If you are eliciting a treatment effect, you should prepare to ask your experts similar questions as well.

#### Step 4. Ethics clearance and pre-registration

Prior to contacting experts, you will need to apply for ethics approval from your institution in order to collect data from experts. This will involve sharing a project statement describing the background and purpose of your elicitation. We have included an example protocol (Supplementary Material 2), although the ethics approval process will vary depending on your institution’s policies. Be sure to contact your ethics approval board well in advance of your scheduled elicitation in order to obtain approval.

Additionally, the FDA recommends pre-registering before conducting an elicitation [50]. Completing the example protocol in Supplementary Material 2 should provide sufficient detail about your elicitation. This protocol should then be uploaded to ClinicalTrials.gov or Open Science Framework before carrying out any elicitation sessions.

#### Step 5. Identify and select your experts

Before identifying experts for your elicitation, it is important to set out criteria defining the qualifications of a potential expert. You might consider how long they have practiced medicine, whether they have treated a specific number of cases of your disease of interest, or whether they have published papers on the topic of your elicitation. Be mindful that these criteria can be poor proxies for the ability to provide quality judgement in an elicitation [51–53]. For instance, Burgman et al. found that publication records and years of experience were poor predictors of accuracy in elicitation [51]. Nonetheless, these attributes can help guide your search for experts, as your experts must possess substantive knowledge in their field [54]. If your trial aims to gather evidence for a specific region, experts with experience practicing in that region would be best suited. Depending on your research question, you may wish to recruit a mix of physicians from community and academic hospitals. Practically, experts also need to speak in the same language as your elicitation. Furthermore, recruiting impartial experts who are not involved in the trial should also be prioritized to improve the quality of the elicited prior. Above all, the IDEA protocol requires that experts are able to understand the elicitation questions.

Next, consider your expert recruitment strategy. One popular method of recruiting clinicians to a clinical trial prior elicitation is to use email lists for societies that specialize in your trial’s disease area [26, 55–58]. For rare diseases, Orphanet’s expert centers search (https://www.orpha.net/en/expert-centres) could also be used to connect with eligible experts, as was used by Hampson (2015) [20].

You should aim to minimize the selection bias in choosing your experts. Experts that are eager to participate in your elicitation may systematically differ from all eligible experts. For instance, interested experts may have stronger opinions in favour or against the intervention in question. Ramanan (2019) implemented a random selection of experts, to reduce selection bias and improve the generalizability of their expert judgements. In practice, the availability and number of eligible experts will impact whether a random selection process is feasible. In any case, you should document why experts decline to participate in your elicitation. You can then assess whether their reasons might be related to their judgements, which would indicate selection bias.

The IDEA protocol recommends recruiting 10–20 experts for your elicitation. To accommodate the availability of your experts, you can split this group and run multiple, smaller sessions. However, each session should contain multiple and varied experts to ensure different viewpoints are represented.

We would also advise budgeting compensation for your experts’ time and specialized knowledge. In exchange for their participation, experts could be provided an honorarium or paid an hourly rate.

#### Step 6. Assemble your evidence

You should gather a dossier of comprehensive evidence on the topic of your elicitation. Your dossier should include trials and observational studies that use similar interventions on similar populations. For ultra-rare diseases, evidence on the broader group of conditions the disease belongs to would be relevant to experts preparing for an elicitation. In the interest of time for a synchronous elicitation session, this evidence dossier should be shared with the experts prior to the session.

#### Step 7. Prepare your workshop materials

In preparation for your workshop, we recommend having your facilitator plan the flow of the session. This involves creating a script for your workshop to ensure that all key components are included and that there is consistent delivery if the workshop is being repeated [13]. We have attached a script from a previous elicitation as a supplement to help with your script (Supplementary Material 3). We also typically prepare a PowerPoint presentation for the session (Supplementary Material 4). Please feel free to reuse and adapt these materials to fit your elicitation.

Another essential component for your workshop is a tool to visualize and collect the elicitation estimates. Your analyst will lead the development of this tool. The goal of visualizing the estimates is to provide experts with a greater understanding of their answers, so that they can calibrate their judgements. For our previous elicitations, we designed RShiny applications to visualize expert estimates, display visualizations, and collect estimates for our elicitation [22, 23]. These tools summarize expert estimates alongside a graphical display of the corresponding distribution. Your analyst will decide on the relevant distribution for your elicitation problem. In addition to a summary and graphical display, it would be helpful to provide experts with an estimated number of patients that their elicitation answers correspond to, which is known as the effective sample size. This has previously been calculated in the context of a rare disease trial elicitation [20]. The effective sample size for different distributions can be analytically calculated or approximated as described here [59]. Because the elicited quantities will be combined with trial data, you should advise the experts to adjust their estimates using the effective sample size to ensure that the prior does not dominate the analysis. To facilitate the design of your visualization tool, we provide a template code base for an RShiny application here (Supplementary Material 5). Generic elicitation software such as MATCH or EXPLICIT could also be suitable for your elicitation [36, 60]. Furthermore, we recommend exploring the available templates on the Structured Expert Elicitation Platform (https://github.com/priorb-source/seep) [61]. Visualization software should be prepared for each elicitation question as well as the practice question mentioned in Step 3.

After designing your workshop and visualization tool, we recommend that you pilot your workshop. Your team should independently rehearse the workshop before inviting an expert to participate and provide feedback on the workshop. Any feedback can then be integrated into the elicitation workshop.

### Elicitation

The section outlines the format for an in-person or virtual, synchronous workshop. Similar approaches can be adopted for an asynchronous elicitation.

#### The workshop

The workshop is a chance for experts to *Investigate*, *Discuss*, and *Estimate* elicitation quantities, as a part of the IDEA protocol. Workshops consist of an introduction, a first round of elicitation questions, a discussion, and a second round of elicitation questions.

During the introduction, you should provide background and rationale for your trial and its associated elicitation. This is an opportunity to build rapport with the experts, so introduce your team members and experts to each other. We also recommend defining any statistical concepts to aid clinicians with the elicitation, such as the concept of a credible interval. Finally, it would be helpful to demo your visualization tool with the example question (Step 3 & 7), to familiarize experts with the elicitation process.

During round 1 (Investigate), experts should individually answer the elicitation questions using the previously described elicitation tool (Step 7). The analyst should then generate boxplots – one for each expert’s set of estimates on each elicitation quantity. The boxplots should be anonymized to reduce the influence of seniority, reputation, or perceived expertise on how participants evaluate the estimates.

During the discussion (Discuss), experts should be presented with all boxplots from their workshop. The facilitator can then ask why their estimates differ, providing an opportunity for experts to voice their interpretation of the elicitation questions and clarify misunderstandings. Experts can also reflect on the additional external information they were presented. The facilitator should manage group dynamics such that dominant personalities do not monopolize the discussion. In our experience, a 15-minute discussion is generally a sufficient opportunity for experts to discuss two quantities.

During round 2 (Estimate), experts are again individually asked to answer the elicitation questions. The responses to this second round of elicitation are used as the final estimates.

To accommodate the availability of the participating experts, this workshop can be repeated on multiple occasions. This has the potential to allow a greater diversity of experts to participate and contribute to the elicitation.

#### Post-workshop

The IDEA protocol prescribes the use of a mathematical aggregation strategy to calculate a collective expert estimate. One approach for combining expert judgements is quantile aggregation. In this method, estimates for the lower, best, and upper values are averaged across experts. While quantile aggregation is recommended in the IDEA protocol, there is mixed evidence as to whether it performs as well as aggregating distributions [62–64]. However, given the simplicity and interpretability of this method, this could be a reasonable option for your elicitation context.

Alternatively, you may opt to generate a mixture of the distributions representing each expert’s knowledge on each quantity with equal-weighted linear pooling. In this method, expert judgements are first fit to a distribution, before samples are drawn from each distribution. These samples are then combined to generate a pooled distribution. This approach may perform better than a quantile aggregation [62, 63], however, can be more complex to implement. To help with the implementation of this method, we have included sample code (Supplementary Material 6).

If you are eliciting the treatment effect, you should also consider whether the quantities you elicit for the control and intervention arms are independent of each other. Given that experts are likely to use one estimate to anchor the other, we suggest that you model the two quantities in a dependent manner. Thall et al. demonstrate an elicitation aggregation, where bivariate distributions are constructed for the control and intervention estimates [25]. The previously described covariates (Step 3) were used to construct latent effects that introduced a correlation between expert’s estimates of the primary outcome response in each arm. Please refer to their code in Supplementary Material 7 in order to apply this method to your elicitation. This approach increases both the accuracy and complexity of the modelling procedure.

After aggregating the estimates, visual summaries of the individual and aggregated estimates should be circulated to the experts. All experts should sign off on any final elicited distributions. After approval, the final aggregate can be used as the prior distribution for your trial. Finally, when reporting your trial, you should ensure that you report all elicitation elements mentioned in the ISPOR Good Practices Task Force guidelines [65].

## Conclusion

This guide outlines the steps needed to conduct an elicitation for a rare disease trial. Given that previous rare disease trials do not use structured elicitation approaches, we have provided a prescriptive set of instructions for implementing the IDEA protocol for rare disease trials.

One limitation of elicitation is that we cannot ensure that elicited beliefs are accurate. This may be in part due to selection biases that may arise when recruiting your experts. As there is limited preexisting data in the rare disease setting, expert estimates typically cannot be verified until you collect trial data. After collecting trial data, we recommend validating expert judgements with prior-data checks that test whether the prior distribution is realistic in comparison to trial data [66]. Your statistical analysis plan should pre-specify a plan for handling prior-data conflict through the use of noninformative prior distributions or the implementation of Bayesian borrowing approaches (see Lesaffre et al. [67]). Although there is a potential for inaccuracy, elicitation is the best option for constructing a prior distribution that represents the current state of knowledge when there are no preexisting data. This is reflected in the recent FDA draft guidance on using Bayesian methods in clinical trials that recommends expert elicitation in certain settings [50]. The acceptability of prior elicitation should be discussed with regulatory bodies during trial planning for studies intended to inform regulatory decision-making.We recommended using the IDEA protocol to develop prior distributions for rare disease trials. We did not explore other protocols such as Sheffield Elicitation Framework (SHELF) or Cooke’s classical method, which may better fit your setting. If you are interested in other structured protocols, we recommend this review [65] as an introduction to other methods. As previously discussed, there is no way of assessing the accuracy of elicited beliefs in the context of limited data. For this reason, we cannot compare the accuracy of the protocols against each other or comment on the quality of the results of the elicitations with different protocols. While there are many elicitation protocol options, we believe that IDEA is best suited to the rare disease trial context. Cooke’s method requires that calibration of expert judgement on seed questions that test experts on known quantities in their domain. Seed questions can be challenging to generate in the rare disease setting where there are few known quantities. SHELF requires a face-to-face meeting to perform behavioural aggregation, the process of combining expert judgements through discussion. This can be prohibitively costly in the rare disease setting, where experts tend to be distributed internationally. The IDEA protocol provides a flexible framework that can be adapted to multiple modes of elicitation.

A future direction for elicitation research is to further develop approaches to visualize estimates and model different treatment effects using bivariate approaches. Methods for constructing bivariate prior distributions for treatment effects have been developed for binary outcomes. Future research should extend these visualization and bivariate methods to survival, continuous, or even complex repeated measure outcomes.

This paper focuses on eliciting the prior distribution for a rare disease trial with the IDEA protocol. It provides practical recommendations for planning and executing the elicitation process. We hope that this guide encourages the use of elicitation, particularly structured elicitation methods, in rare disease trials. These guidelines will allow investigators to construct valid, reproducible prior distributions for the treatment effect of interest in future rare disease trials.

## Supplementary Information

Below is the link to the electronic supplementary material.

Supplementary Material 1

Supplementary Material 2

Supplementary Material 3

Supplementary Material 4

Supplementary Material 5

Supplementary Material 6

Supplementary Material 7

## Data Availability

No datasets were generated or analysed during the current study.

## Abbreviations

IDEA: Investigate Discuss Estimate Aggregate
SHELF: Sheffield Elicitation Framework
