# Supplementary material for: A primer on expert elicitation with IDEA (Investigate Discuss Estimate Aggregate) for rare disease trialists
Source: J Rare Dis (Berlin). 2026 Nov 4;5(1):111. doi: 10.1007/s44162-026-00253-8 (PMC13736339; doi:10.1007/s44162-026-00253-8)
Supplement: Supplementary file 1 [file 44162_2026_253_MOESM1_ESM.docx]

# Supplementary Material 1

The following examples illustrate scenarios and questions in rare diseases of varying prevalence that could be used for your elicitation.

You are interested in eliciting a prior distribution for your trial comparing mesenchymal stromal cells to standard of care for preventing bronchopulmonary dysplasia. Given that MSCs are only used in clinical trials, your experts do not have experience with MSCs. Therefore, you opt to elicit the outcome in the comparator arm only.

Scenario 1:

Consider a preterm male infant born at 24-week gestation with a birth weight of 600g. He is now three weeks old on jet ventilation with FiO2 requirements around 35%. He was born to a 31-year-old mother with an uncomplicated pregnancy until she was admitted for vaginal bleeding at 24 weeks for which she required an emergency C-section for abruption. No antenatal steroids were given. He was intubated on first attempt in the delivery room and received 2 doses of surfactant at one and six hours of life. He was treated with indomethacin for a large patent ductus arteriosus. He developed a stage 2 necrotizing enterocolitis at day 7 of life for which he was treated with antibiotics and kept NPO for 10 days. He is tolerating slow feed increases through an oral gastric tube and receives parenteral nutrition through a central IV line. You identify this patient at high risk of developing severe bronchopulmonary dysplasia (BPD).

You opt to treat your patients using the current standard of care, which includes an attempt at extubation to non-invasive ventilation as early as possible and caffeine treatment.

Question Set 1:

- Of 20 similar patients, what is the lower plausible value for the number of patients who die or develop BPD by 36 weeks?
- Of 20 similar patients, what is the upper plausible value for the number of patients who die or develop BPD by 36 weeks?
- Of 20 similar patients, what is the most likely value for the number of patients who die or develop BPD by 36 weeks?
- What confidence do you have that the value lies between your lowest and highest plausible values? Pick a value between 50-100%.

Your goal is to conduct a trial comparing semaglutide to Orforglipron on patients with hypothalamic obesity. The following scenario and questions will be used for your elicitation of the prior distribution.

Scenario 2:

A 14 year old status post craniopharyngioma surgery (age of 10) is seen in clinic for follow up of panhypopituitarism. She is receiving hormonal replacement for hypothyroidism, secondary gonadal failure, growth hormone deficiency, ACTH deficiency and diabetes insipidus. There has been an alarming gain of weight (BMI 34; weight 100kg;Ht 160cm) that cannot be attributed to her underlying hormonal deficits nor nutritional excess. Considering the history and previous surgery, you diagnose hypothalamic obesity. Given the high risk of morbidity and mortality, you decide on treatment with a GLP-1 receptor agonist.

Question Set 2:

Based on case studies, you choose to treat with injectable Semaglutide at a dose of 1.7 mg once weekly.

- Of 20 similar patients, what is the lower plausible value for the number of patients who will benefit from stabilization or reduction of weight at 3 months?
- Of 20 similar patients, what is the upper plausible value for the number of patients who will benefit from stabilization or reduction of weight at 3 months?
- Of 20 similar patients, what is the most likely value for the number of patients who will benefit from stabilization or reduction of weight at 3 months?
- What confidence do you have that the value lies between your lowest and highest plausible values? Pick a value between 50-100%.

Based on case studies, you choose an oral GLP-1 (Orforglipron) at a dose of 12 mg.

- Of 20 similar patients, what is the lower plausible value for the number of patients who will benefit from stabilization or reduction of weight at 3 months?
- Of 20 similar patients, what is the upper plausible value for the number of patients who will benefit from stabilization or reduction of weight at 3 months?
- Of 20 similar patients, what is the most likely value for the number of patients who will benefit from stabilization or reduction of weight at 3 months?
- What confidence do you have that the value lies between your lowest and highest plausible values? Pick a value between 50-100%.

You are conducting a trial comparing adenine base editing gene therapy to the standard of care, hematopoietic stem cell transplantation (HCT), for CD3d severe combined immune deficiency (SCID). Outcomes of HCT for SCID are complex due to the use of a variety of protocols and variables for transplant. Prior clinical trials for gene therapies for other types of SCID have been conducted as phase I/II trials with no controls. Historical control data (patients treated with HCT) have not been utilized to formally compare outcomes of gene therapy to transplant in this population. As a result, you opt to utilize expert elicitation to determine outcome for the control and comparator arms. Given that the outcome is likely to among the higher range of the values, the following questions are framed around 100 patients, rather than 20 patients.

Scenario 3:

A 10-day old baby has been identified to have an abnormal newborn screen result for SCID. The baby is urgently referred to Immunology. The baby is isolated and started on prophylactic therapies to prevent infections. Further investigations confirm the baby has SCID caused by a founder pathogenic variant in the CD3D gene. The baby does not have a matched sibling donor for transplant; however, a haploidentical transplant could be performed using a parent as the donor. The patient could either be treated with HCT or enrolled on a gene therapy trial for CD3d SCID.

Question Set 3:

For the questions, 2-year event free survival (EFS) is defined as: survival; no need for a second definitive therapy (HCT or gene therapy); no need for either donor lymphocyte infusions or stem cell boosts for poor engraftment; no acute graft versus host disease above grade II; no moderate or severe chronic graft versus host disease.

Based on case studies for similar patients, you choose to treat the baby with HCT:

- Of 100 similar patients, what is the lower plausible value for the number of patients who will have a 2-year event free outcome following HCT?
- Of 100 similar patients, what is the upper plausible value for the number of patients who will have a 2-year event free outcome following HCT?
- Of 100 similar patients, what is the most likely value for the number of patients who will have a 2-year event free outcome following HCT?

Based on case studies of gene therapy for other types of SCID, you choose to enroll the baby on the gene therapy clinical trial:

- Of 100 similar patients, what is the lower plausible value for the number of patients who will have a 2-year event free outcome following gene therapy?
- Of 100 similar patients, what is the upper plausible value for the number of patients who will have a 2-year event free outcome following gene therapy?
- Of 100 similar patients, what is the most plausible value for the number of patients who will have a 2-year event free outcome following gene therapy?

You are conducting an ultra-rare disease trial that requires prior distributions for the outcome (Question Set 4A) and the variability in the outcome (Question Set 4B).

Scenario 4:

A seven-year-old child has an ultra-rare genetic condition resulting in a severe intellectual disability, uncoordinated motor movements and hypotonia, cortical visual impairment, and infrequent seizures (focal seizures 2-3 times per year with viral illnesses). This child cannot communicate verbally, nor with gestures, and relies on a gastrostomy tube for nutrition given risk of aspiration. They can walk several steps if supported on both sides and can pick up and transfer an object between each hand. Their parents describe them as having progressed very slowly in their development to this point, with no one period of regression or loss of skills. This child was tested on the Bayley Scales of Infant and Toddler Development and the Vineland Scales of Adaptive Behavior which found their total composite scores on both measures to be below the first percentile (severe delays). The family has prioritized motor skill development as the treatment outcome of interest. On both of these measures, their age equivalent scores at 7 years suggested they had motor skills comparable to a 12-month-old child.

Question Set 4A: Eliciting a range of possible values

- If this patient were to be retested on this measure **2 years later** (at age 9 years), assuming there is no known research or clinical evidence regarding their expected disease trajectory available, what is the **lowest plausible value** (age equivalent **score in months**) that you would expect for their **motor** skill development?
- If this patient were to be retested on this measure **2 years later** (at age 9 years), assuming there is no known research or clinical evidence regarding their expected disease trajectory available, what is the **highest plausible** value (age equivalent **score in months**) that you would expect for their **motor** skill development?
- If this patient were to be retested on this measure 2 years later (at age 9 years), assuming there is no known research or clinical evidence regarding their expected disease trajectory available, what is the **most likely** value (age equivalent **score in months**) that you would expect for their **motor skill** development?
- What confidence do you have that the value lies between your lowest and highest plausible values? Pick a value between 50-100%.

Question Set 4B: Heterogeneity

- Out of 20 similar patients who were then retested 2 years later using these measures (at age 9 years), assuming no known research or clinical evidence regarding their expected disease trajectory is available, **how many would you expect to make little to no gains** in developmental progress during this two year time period (i.e., motor skills age equivalent scores in the 12 to 18 month range), assuming no disease modifying treatment was available?
- Out of 20 similar patients who were then retested 2 years later using these measures (at age 9 years), assuming no known research or clinical evidence regarding their expected disease trajectory is available, **how many would you expect to make moderate gains** in developmental progress during this two year time period (i.e., motor subscale age equivalent scores are now in the 18 to 30 month old range), assuming no disease modifying treatment was available?
- Out of 20 similar patients who were then retested 2 years later using these measures (at age 9 years), assuming no known research or clinical evidence regarding their expected disease trajectory is available, how many would you expect **to make large gains in developmental progress during** this two year time period (i.e., motor subscale age equivalent scores are now estimated at 30 months or greater), assuming no disease modifying treatment was available?
- What confidence do you have that the value lies between your lowest and highest plausible values? Pick a value between 50-100%.
