# Supplementary material for: A primer on expert elicitation with IDEA (Investigate Discuss Estimate Aggregate) for rare disease trialists
Source: J Rare Dis (Berlin). 2026 Nov 4;5(1):111. doi: 10.1007/s44162-026-00253-8 (PMC13736339; doi:10.1007/s44162-026-00253-8)
Supplement: Supplementary file 2 [file 44162_2026_253_MOESM2_ESM.docx]

Slide 1

Hi everyone, welcome to the elicitation workshop for the [trial name].

My name is [insert facilitator name] – I will be your workshop facilitator alongside our analyst, [insert analyst name] for today. [Describe that workshop will be repeated, if appropriate].

Slide 2

The [trial name] is a [describe your trial]. The purpose of this workshop is to formulate a prior probability distribution that will be used for the design and analysis of the [trial name].

Slide 3

In place of standard frequentist statistics, this trial will implement Bayesian methods. Frequentist trial designs require assumptions for significance (think less than 5% error), power (80% is typically deemed acceptable), and an effect size of interest. These assumptions are complex and prone to error given that there is a need for a trial and limited knowledge in these areas.

Bayesian statistics incorporate prior information into analyses. This prior information can come in the form of data or expert opinion. In this case, prior information will be derived from your expert opinion and experience in [disease area]. We will aggregate your individual distribution to form a prior distribution, to represent all of your opinions. These aggregated expert opinions will form prior probabilities that will be used to the [trial name], using Bayesian methods.

Unshare Screen (if facilitated online)

Today we have [number of experts] experts from [region(s)]. Could we start with a round of introductions?

Thanks so much for your time - we really appreciate your participation.

Share Screen – slide 5

The workshop will consist of two rounds of surveys, separated by a short break and a group discussion. Each survey round will include X questions to elicit your beliefs. We will also collect information about your professional experience through a short questionnaire.

Today’s workshop involves minimal risk. As a benefit, you will gain more knowledge in the treatment of [disease] and will be provided with study results to see the quantitative expression of your subjective judgement. We hope that the information learned from this study can be used in the future to benefit other researchers who are aiming to understand how treatments for [disease] are viewed by experts. The output from this study will also be used to support the design and analysis of the [trial name], which will generate evidence regarding the treatment of [population] with [disease].

If you have any questions about this project, please get in touch with our team, we are very happy to provide any additional information. By completing these questionnaires, you are consenting to its use in research.

All information collected about you will be “de-identified” by replacing your identifiable information (i.e., name) with a “study number”. Only the “study code key” can connect the information collected about you to your identity. The study code key will be safeguarded by the research team at [research institute]. Even though the risk of identifying you from the study data is very small, it can never be completely eliminated.

Slide 6

Prior to today’s workshop, you should have received an email containing the materials for today’s workshop. You should have received a link to the elicitation tool and our professional experience questionnaire [if using bivariate elicitation]. The elicitation tool can only be accessed using a computer or laptop.

Slide 7

Our workshop will primarily be hosted on the Elicitation Tool on R-Shiny Dashboard. The menu on the left-hand side will guide you through each survey round.

Slide 8

Each round will consist of X questions.

Slide 9

Here is a sample question page.

Slide 10

*Read slide verbatim*

Unshare Slides/Share Shiny App

Let’s go through an example. After reading the instructions, let’s review the question.

You're a tech product manager, and you're deciding between two email notification frequencies for a mobile app.

Given the standard notification frequency of once a day. Out of 100 app users who receive daily email notifications, how many will unsubscribe within a week due to email overload?

First let’s choose the lower and upper plausible values for the number of people who unsubscribe.

Based on my experience, I tend to hear of people receiving too many emails, incentivizing unsubscribing. On the other hand, people may not unsubscribe for a variety of reasons: maybe they have email filters, or do not check their email frequently. Furthermore, I will consider that we would be interested in people unsubscribing within a week for this question. Based on these factors, I will select 1 as the lower plausible value and 40 as the upper plausible value. Next, I’ll select the most likely value. Notice how adjusting the slider changes which answers are most probable. I think a most likely value of 7 best represents my beliefs. I have 90% confidence that the value lies between my upper and lower estimates. Now, after reviewing the output, I believe I have adequately captured my beliefs.

------------------------------------------------------------------------------------------------------------------------------

Slide 11

The formal elicitation process will be presented in a similar format. Once everyone has completed and submitted their answers for both questions, there will be a short break. If you have not already, please use this time to fill out the professional experience survey and take some time away from your screen. Our analyst will generate a deidentified boxplot with everyone’s answers that will be discussed for 10-15 minutes. Please share your thoughts with other group members. Remember that it is not necessary to reach a consensus across the group, instead use the boxplot and discussion to calibrate your judgements. In the second round, you may adjust your answers.

Slide 12

We will begin the formal elicitation now. Please open the Elicitation Tool link. Is everyone able to open step 1 of round 1? Ok, please get started with the round of questions. You will have 8 minutes to complete the round. If you finish early, please use this time to fill out the professional experience questionnaire. Please let me know if you have any questions.

Slide 13

We’re now moving into the discussion stage. You should have completed the professional experience questionnaire at this time as well [if using bivariate elicitation]. During this stage, we will be presenting a boxplot for each answer. Each boxplot will display a median (50% of patients are under this value and 50% over). The two lines represent whiskers. Longer whiskers represent more conservative opinions.

[Discuss for 15 minutes – optional seed questions below]

- What information did you draw on to select your central tendency?
- What decision-making process led you to choose your estimates?
- What factors may have introduced different levels of uncertainty in the distributions?
- If you are willing to share which distribution you produced, why do you think yours differs from the others presented?

Slide 14

Let’s move on to our last step here the second-round questionnaire. Please navigate back to the Elicitation Tool. If the page has disconnected, please refresh the page.

This second-round will present the same questions as round 1. However, these answers will be used in our final analyses. You may change your answers from round 1. Please let me know if you have any questions, and you may start the second round now.

Page 15

It looks like everyone has completed the final questionnaire. We will circulate an individual and group distribution two weeks after the (final) workshop. Please also let our team know if you do not wish to be acknowledged in the final publication. If you have any questions, please feel free to email the team.

Thanks so much for your participation and discussion today.
