# Supplementary material for: A primer on expert elicitation with IDEA (Investigate Discuss Estimate Aggregate) for rare disease trialists
Source: J Rare Dis (Berlin). 2026 Nov 4;5(1):111. doi: 10.1007/s44162-026-00253-8 (PMC13736339; doi:10.1007/s44162-026-00253-8)
Supplement: Supplementary file 3 [file 44162_2026_253_MOESM3_ESM.docx]

\# The following code can be used to visualize a binary primary outcome.

\# Please adopt the following code to fit your elicitation setting. We have

\# tagged areas that must be modified with a "# Modify" comment, but feel free

\# to make any other changes

library(shiny)

library(ggplot2)

library(bs4Dash)

library(prevalence)

\# Helper function

adjust_value <- function(value) {

if (value == 0) {

return (.Machine$double.eps)

}

if (value == 1) {

return (1-.Machine$double.eps)

}

return (value)

}

\# Shiny Code

ui <- dashboardPage(

dark=NULL,

help=NULL,

title="Elicitation",

header=dashboardHeader(

dashboardBrand(

title="Elicitation Tool"

)

),

sidebar=dashboardSidebar(

sidebarMenu(

id = "sidebarMenuid",

menuItem(

"Home",

tabName = "home",

icon = icon("home")

),

menuItem(

"Example",

tabName = "example",

icon = icon("magnifying-glass")

),

menuItem(

"Round 1",

tabName = "r1",

icon=icon("question"),

menuSubItem (

"Question 1", tabName="r1q1", icon=icon("circle")),

menuSubItem (

"Question 2", tabName="r1q2", icon=icon("circle"))

),

menuItem(

"Group Discussion",

tabName = "discussion",

icon=icon("book-open")

),

menuItem(

"Round 2",

tabName = "r2",

icon=icon("question"),

menuSubItem (

"Question 1", tabName="r2q1", icon=icon("circle")),

menuSubItem (

"Question 2", tabName="r2q2", icon=icon("circle"))

)

)

),

body=dashboardBody(

tabItems(

tabItem(

tabName="home",

jumbotron(

title="Welcome to the Elicitation for ...", # Modify

lead="", # Modify with any additional background

list(

tags$li("Facilitator: ..."), # Modify

tags$li("Analyst: ..."), # Modify

tags$li("Coordinator: ..."), # Modify

tags$li("Principal Investigator: ...")), # Modify

status="info",

btnName=NULL

)

),

tabItem(

tabName = "example",

fluidRow(

bs4Card(

title="Instructions",

status="primary",

solidHeader = FALSE,

collapsible = TRUE,

collapsed=FALSE,

closable = FALSE,

labelStatus = "primary",

labelText = "",

width = 12,

h4(""), # Modify title

tags$ol(

tags$li("Use the slider to answer the following questions"),

tags$li("The plot section will transform you answers into a summary and

a probability distribution that describe your opinion based on your input"),

tags$li("If the summary and the distribution cannot represent

your opinion, please change your input until

they matched"),

tags$li("If you are satisfied with the summary and distribution,

please submit your answer and move to the next question"),

tags$li("If you have any questions, please feel free to ask the facilitator")) # tags END

),

fluidRow(

column(

width = 6,

bs4Card(

title="Question",

status = "primary",

solidHeader = FALSE,

collapsible = FALSE,

collapsed=FALSE,

closable = FALSE,

labelStatus = "primary",

labelText = "",

width = 12,

fluidRow(

bs4Callout(

title=NULL,

width = 12,

elevation = 1,

status = "info",

tags$p("You're a financial planner with years of experience in managing different sized portfolios."), # Modify

tags$p("Out of 20 lottery winners, how many will spend all of their winnings within two years?") # Modify

) # callout end

), # fluid row end

fluidRow(

column(

width=12,

\# Step1

tags$strong("Step 1:"),

tags$p( "Based on your expertise and experience, please use the slider to choose the", tags$strong("LOWER

and UPPER plausible values"), "for the question above."),

sliderInput(inputId = "examplerange",label="",value=c(5, 15),min=0,max=20,width="100%"),

\# Step2

tags$strong("Step 2:"),

tags$p("Based on your expertise and experience, please use the slider to choose the", tags$strong("BEST

or MOST LIKELY value"), "for the question above."),

tags$code("The BEST/ MOST LIKELY value CANNOT be greater than the UPPER plausible value and smaller

than then the LOWER plausible value. "),

sliderInput(inputId = "exampleestimate",label="",value=10,min=5,max=15,width="100%"),

\# Step 3

tags$strong("Step 3:"),

tags$p("What confidence do you have that the value lies between your

lower and upper estimates? Pick a value between 50 to 100%."),

sliderInput(inputId = "exampleconfidence", label="", value=75, min=50, max=100, width="100%")

)

)

)

),

column(

width=6,

bs4Card(

title="Response",

status="primary",

solidHeader = FALSE,

collapsible = FALSE,

collapsed=FALSE,

closable = FALSE,

labelStatus = "primary",

labelText = "",

width = 12,

fluidRow(

callout(

title="Summary",

width = 12,

elevation=1,

status = "info",

htmlOutput("summaryexample")

)

), #

fluidRow(

callout(

title="Effective Sample Size",

width = 12,

elevation=1,

status = "info",

htmlOutput("essexample")

)

),#

plotOutput("plotexample")

)

)

)

)

),

\#

tabItem(

tabName = "r1q1",

fluidRow(

bs4Card(

title="Instructions",

status="primary",

solidHeader = FALSE,

collapsible = TRUE,

collapsed=FALSE,

closable = FALSE,

labelStatus = "primary",

labelText = "",

width = 12,

h4(""), # Modify title

tags$ol(

tags$li("Use the slider to answer the following questions"),

tags$li("The plot section will transform you answers into a summary and

a probability distribution that describe your opinion based on your input"),

tags$li("If the summary and the distribution cannot represent

your opinion, please change your input until

they matched"),

tags$li("If you are satisfied with the summary and distribution,

please submit your answer and move to the next question"),

tags$li("If you have any questions, please feel free to ask the facilitator")) # tags END

),

fluidRow(

column(

width = 6,

bs4Card(

title="Question",

status = "primary",

solidHeader = FALSE,

collapsible = FALSE,

collapsed=FALSE,

closable = FALSE,

labelStatus = "primary",

labelText = "",

width = 12,

fluidRow(

bs4Callout(

title=NULL,

width = 12,

elevation = 1,

status = "info",

tags$p("[Insert case study]"), # Modify

tags$p("[Insert elicitation question] ") # Modify

) # callout end

), # fluid row end

fluidRow(

column(

width=12,

\# Step1

tags$strong("Step 1:"),

tags$p( "Based on your expertise and experience, please use the slider to choose the", tags$strong("LOWER

and UPPER plausible values"), "for the question above."),

sliderInput(inputId = "round1range1",label="",value=c(5,15),min=0,max=20,width="100%"),

\# Step2

tags$strong("Step 2:"),

tags$p("Based on your expertise and experience, please use the slider to choose the", tags$strong("BEST

or MOST LIKELY value"), "for the question above."),

tags$code("The BEST/ MOST LIKELY value CANNOT be greater than the UPPER plausible value and smaller

than then the LOWER plausible value. "),

sliderInput(inputId = "round1estimate1",label="",value=10,min=5,max=15,width="100%"),

\# Step 3

tags$strong("Step 3:"),

tags$p("What confidence do you have that the value lies between your

lower and upper estimates? Pick a value between 50 to 100%."),

sliderInput(inputId = "round1confidence1", label="", value=75, min=50, max=100, width="100%")

)

)

)

),

column(

width=6,

bs4Card(

title="Response",

status="primary",

solidHeader = FALSE,

collapsible = FALSE,

collapsed=FALSE,

closable = FALSE,

labelStatus = "primary",

labelText = "",

width = 12,

fluidRow(

callout(

title="Summary",

width = 12,

elevation=1,

status = "info",

htmlOutput("summaryr1q1")

)

), #

fluidRow(

callout(

title="Effective Sample Size",

width = 12,

elevation=1,

status = "info",

htmlOutput("essr1q1")

)

),#

plotOutput("plotr1q1")

)

)

)

)

),

tabItem(

tabName = "r1q2",

fluidRow(

bs4Card(

title="Instructions",

status="primary",

solidHeader = FALSE,

collapsible = TRUE,

collapsed=FALSE,

closable = FALSE,

labelStatus = "primary",

labelText = "",

width = 12,

h4(""), # Modify title

tags$ol(

tags$li("Use the slider to answer the following questions"),

tags$li("The plot section will transform you answers into a summary and

a probability distribution that describe your opinion based on your input"),

tags$li("If the summary and the distribution cannot represent

your opinion, please change your input until

they matched"),

tags$li("If you are satisfied with the summary and distribution,

please submit your answer and move to the next question"),

tags$li("If you have any questions, please feel free to ask the facilitator")) # tags END

),

fluidRow(

column(

width = 6,

bs4Card(

title="Question",

status = "primary",

solidHeader = FALSE,

collapsible = FALSE,

collapsed=FALSE,

closable = FALSE,

labelStatus = "primary",

labelText = "",

width = 12,

fluidRow(

bs4Callout(

title=NULL,

width = 12,

elevation = 1,

status = "info",

tags$p("[Insert case study]"), # Modify

tags$p("[Insert elicitation question] ") # Modify

)

),

fluidRow(

column(

width=12,

\# Step1

tags$strong("Step 1:"),

tags$p( "Based on your expertise and experience, please use the slider to choose the", tags$strong("LOWER

and UPPER plausible values"), "for the question above."),

sliderInput(inputId = "round1range2",label="",value=c(5,15),min=0,max=20,width="100%"),

\# Step2

tags$strong("Step 2:"),

tags$p("Based on your expertise and experience, please use the slider to choose the", tags$strong("BEST

or MOST LIKELY value"), "for the question above."),

tags$code("The BEST/ MOST LIKELY value CANNOT be greater than the UPPER plausible value and smaller

than then the LOWER plausible value. "),

sliderInput(inputId = "round1estimate2",label="",value=10,min=5,max=15,width="100%"),

\# Step 3

tags$strong("Step 3:"),

tags$p("What confidence do you have that the value lies between your

lower and upper estimates? Pick a value between 50 to 100%."),

sliderInput(inputId = "round1confidence2", label="", value=75, min=50, max=100, width="100%")

)

)

)

),

column(

width=6,

bs4Card(

title="Response",

status="primary",

solidHeader = FALSE,

collapsible = FALSE,

collapsed=FALSE,

closable = FALSE,

labelStatus = "primary",

labelText = "",

width = 12,

fluidRow(

callout(

title="Summary",

width = 12,

elevation=1,

status = "info",

htmlOutput("summaryr1q2")

)

),

fluidRow(

callout(

title="Effective Sample Size",

width = 12,

elevation=1,

status = "info",

htmlOutput("essr1q2")

)

),

plotOutput("plotr1q2")

)

)

)

)

),

\# Group Discussion

tabItem(

tabName = "discussion",

fluidRow(

bs4Card(

title="Instructions",

#status="info",

solidHeader = FALSE,

collapsible = TRUE,

collapsed=FALSE,

closable = FALSE,

labelStatus = "primary",

labelText = "",

width = 12,

h4("Welcome to the group discussion section"),

tags$ol(

tags$li("Read the following summaries and probability distributions that

are generated from the first round input"),

tags$li("If you want to make a change to your answer and adjust the summary

and distribution, you will have the chance to revise them in the second round"),

tags$li("Feel free to unmute yourself and share your opinion on the treatments of Bronchiolitis"),

tags$li("Remeber it is not necessary to make an agreement any questions")

)

)),

fluidRow(

bs4Card(

title="Review",

#status = "info",

solidHeader = FALSE,

collapsible = TRUE,

collapsed=FALSE,

closable = FALSE,

labelStatus = "primary",

labelText = "",

width = 12,

fluidRow(

column(

width = 12,

callout(

title=NULL,

tags$p("[Insert case study]"), # Modify

width = 12,

elevation = 1,

status = "info"

)

)

),

fluidRow(

column(

width = 12,

callout(

title="Question 1",

tags$p("[Insert elicitation question]"), # Modify

width = 12,

elevation = 1,

status = "info",

fluidRow(

column(

width=6,

bs4Callout(

status="info",

title="Your Response",

width=12,

htmlOutput("discussionredcap1"),

htmlOutput("discussiontext1"),

htmlOutput("discussioness1")

)),

column(

width=6,

plotOutput("discussionplot1"))

)

)

)

),

fluidRow(

column(

width=12,

callout(

title="Question 2",

tags$p("[Insert elicitation question]"), # Modify

width = 12,

elevation = 1,

status = "info",

fluidRow(

column(

width=6,

bs4Callout(

status="info",

title="Your Response",

width=12,

p(htmlOutput("discussionredcap2")),

p(htmlOutput("discussiontext2")),

htmlOutput("discussioness2"))),

column(

width=6,

plotOutput("discussionplot2"))

)

)

)

)

)

)

),

\# Round 2

tabItem(

tabName = "r2q1",

fluidRow(

bs4Card(

title="Instructions",

status="primary",

solidHeader = FALSE,

collapsible = TRUE,

collapsed=FALSE,

closable = FALSE,

labelStatus = "primary",

labelText = "",

width = 12,

h4(""), # Modify title

tags$ol(

tags$li("Use the slider to answer the following questions"),

tags$li("The plot section will transform you answers into a summary and

a probability distribution that describe your opinion based on your input"),

tags$li("If the summary and the distribution cannot represent

your opinion, please change your input until

they matched"),

tags$li("If you are satisfied with the summary and distribution,

please submit your answer and move to the next question"),

tags$li("If you have any questions, please feel free to ask the facilitator")) # tags END

),

fluidRow(

column(

width = 6,

bs4Card(

title="Question",

status = "primary",

solidHeader = FALSE,

collapsible = FALSE,

collapsed=FALSE,

closable = FALSE,

labelStatus = "primary",

labelText = "",

width = 12,

fluidRow(

bs4Callout(

title=NULL,

width = 12,

elevation = 1,

status = "info",

tags$p("[Insert case study]"), # Modify

tags$p("[Insert elicitation question] ") # Modify

) # callout end

), # fluid row end

fluidRow(

column(

width=12,

\# Step1

tags$strong("Step 1:"),

tags$p( "Based on your expertise and experience, please use the slider to choose the", tags$strong("LOWER

and UPPER plausible values"), "for the question above."),

sliderInput(inputId = "round2range1",label="",value=c(5,15),min=0,max=20,width="100%"),

\# Step2

tags$strong("Step 2:"),

tags$p("Based on your expertise and experience, please use the slider to choose the", tags$strong("BEST

or MOST LIKELY value"), "for the question above."),

tags$code("The BEST/ MOST LIKELY value CANNOT be greater than the UPPER plausible value and smaller

than then the LOWER plausible value. "),

sliderInput(inputId = "round2estimate1",label="",value=10,min=5,max=15,width="100%"),

\# Step 3

tags$strong("Step 3:"),

tags$p("What confidence do you have that the value lies between your

lower and upper estimates? Pick a value between 50 to 100%."),

sliderInput(inputId = "round2confidence1", label="", value=75, min=50, max=100, width="100%")

)

)

)

),

column(

width=6,

bs4Card(

title="Response",

status="primary",

solidHeader = FALSE,

collapsible = FALSE,

collapsed=FALSE,

closable = FALSE,

labelStatus = "primary",

labelText = "",

width = 12,

fluidRow(

callout(

title="Summary",

width = 12,

elevation=1,

status = "info",

htmlOutput("summaryr2q1")

)

),

fluidRow(

callout(

title="Effective Sample Size",

width = 12,

elevation=1,

status = "info",

htmlOutput("essr2q1")

)

),

plotOutput("plotr2q1")

)

)

)

)

),

tabItem(

tabName = "r2q2",

fluidRow(

bs4Card(

title="Instructions",

status="primary",

solidHeader = FALSE,

collapsible = TRUE,

collapsed=FALSE,

closable = FALSE,

labelStatus = "primary",

labelText = "",

width = 12,

h4(""), # Modify title

tags$ol(

tags$li("Use the slider to answer the following questions"),

tags$li("The plot section will transform you answers into a summary and

a probability distribution that describe your opinion based on your input"),

tags$li("If the summary and the distribution cannot represent

your opinion, please change your input until

they matched"),

tags$li("If you are satisfied with the summary and distribution,

please submit your answer and move to the next question"),

tags$li("If you have any questions, please feel free to ask the facilitator")) # tags END

),

fluidRow(

column(

width = 6,

bs4Card(

title="Question",

status = "primary",

solidHeader = FALSE,

collapsible = FALSE,

collapsed=FALSE,

closable = FALSE,

labelStatus = "primary",

labelText = "",

width = 12,

fluidRow(

bs4Callout(

title=NULL,

width = 12,

elevation = 1,

status = "info",

tags$p("[Insert case study]"), # Modify

tags$p("[Insert elicitation question] ") # Modify

)

),

fluidRow(

column(

width=12,

\# Step1

tags$strong("Step 1:"),

tags$p( "Based on your expertise and experience, please use the slider to choose the", tags$strong("LOWER

and UPPER plausible values"), "for the question above."),

sliderInput(inputId = "round2range2",label="",value=c(5,15),min=0,max=20,width="100%"),

\# Step2

tags$strong("Step 2:"),

tags$p("Based on your expertise and experience, please use the slider to choose the", tags$strong("BEST

or MOST LIKELY value"), "for the question above."),

tags$code("The BEST/ MOST LIKELY value CANNOT be greater than the Upper plausible value and smaller

than then the LOWER plausible value. "),

sliderInput(inputId = "round2estimate2",label="",value=10,min=5,max=15,width="100%"),

\# Step 3

tags$strong("Step 3:"),

tags$p("What confidence do you have that the value lies between your

lower and upper estimates? Pick a value between 50 to 100%."),

sliderInput(inputId = "round2confidence2", label="", value=75, min=50, max=100, width="100%")

)

)

)

),

column(

width=6,

bs4Card(

title="Response",

status="primary",

solidHeader = FALSE,

collapsible = FALSE,

collapsed=FALSE,

closable = FALSE,

labelStatus = "primary",

labelText = "",

width = 12,

fluidRow(

callout(

title="Summary",

width = 12,

elevation=1,

status = "info",

htmlOutput("summaryr2q2")

)

),

fluidRow(

callout(

title="Effective Sample Size",

width = 12,

elevation=1,

status = "info",

htmlOutput("essr2q2")

)

),

plotOutput("plotr2q2")

)

)

)

)

)

\#

)

)

)

server <- function(input,output, session) {

\# Example slider input update

observe({

updateSliderInput(session = session, "exampleestimate", value = round((input$examplerange[1]+input$examplerange[2])/2,0),

min = input$examplerange[1],

max=input$examplerange[2])})

\# Example Summary

exampletext<-reactive({

example_lower<-input$examplerange[1]/20

example_upper<-input$examplerange[2]/20

example_estimate<-input$exampleestimate/20

HTML(paste0("Based on your input, out of 20 lottery winners ,",

"<b>", " there is ", input$exampleconfidence, "% probability that the number

of individuals who will spend all their winnings within two years will be between ",

"<u>",input$examplerange[1]," and ", input$examplerange[2],"</u>", ",

and ","<u>", input$exampleestimate,"</u>", " will mostly likely

spend all their winnings within two years.","</b>")) # Modify

})

output$summaryexample<-renderUI(exampletext())

essexampletext <- reactive({

example_lower<-adjust_value(input$examplerange[1]/20)

example_upper<-adjust_value(input$examplerange[2]/20)

example_estimate<-adjust_value(input$exampleestimate/20)

example_betaparam<-betaExpert(example_estimate,example_lower,example_upper,p=input$exampleconfidence/100,

method="mode")

example_a<-example_betaparam$alpha

example_b<-example_betaparam$beta

example_ess <- round(example_a+example_b, 2)

HTML(paste0("Your effective sample size (ESS) is ", "<b><u>", example_ess,

"</b></u>."))

})

output$essexample <- renderUI(essexampletext())

\# Example Plot

exampleplot<-reactive({

example_lower<-adjust_value(input$examplerange[1]/20)

example_upper<-adjust_value(input$examplerange[2]/20)

example_estimate<-adjust_value(input$exampleestimate/20)

example_betaparam<-betaExpert(example_estimate,example_lower,example_upper,p=input$exampleconfidence/100,

method="mode")

example_a<-example_betaparam$alpha

example_b<-example_betaparam$beta

example_p<-seq(0,1,length=100)

exampledata<-data.frame(x=example_p,y=dbeta(example_p,example_a,example_b))

ggplot(data=exampledata,aes(x,y))+geom_line(color="black",size=1)+

labs(title = "Distribution for the probability of spending winnings

within 2 years",x="",y="")+ # Modify

scale_x_continuous(breaks=c(0,0.2,0.4,0.6,0.8,1),labels=c(0,0.2,0.4,0.6,0.8,1))+

theme(plot.title = element_text(size=14, hjust = 0.5))+

theme(panel.border = element_blank())+

theme(axis.text.x = element_text(size=12,face="bold"))+

theme(axis.line.x = element_line(size=1,colour="black"))+

theme(axis.title.x = element_text(size=14))+

theme(axis.ticks.y=element_blank(),axis.text.y = element_blank())+

geom_area(data=subset(exampledata,x>=qbeta(0.025,example_a,example_b)&

x<=qbeta(0.975,example_a,example_b)),aes(x=x,y=y),colour="black",fill="lightblue",alpha=1)

})

output$plotexample<-renderPlot(exampleplot())

\# Round 1 Question 1 slider input update

observe({

updateSliderInput(session = session, "round1estimate1", value = round((input$round1range1[1]+input$round1range1[2])/2,0), min = input$round1range1[1],

max=input$round1range1[2])})

\# Round 1 Question 1 Summary

r1q1text<-reactive({

round1q1_lower<-input$round1range1[1]/20

round1q1_upper<-input$round1range1[2]/20

round1q1_estimate<-input$round1estimate1/20

HTML(paste0("Based on your input, out of 20 similiar patients with [disease] and treated with [comparator]

,","<b>", " there is ", input$round1confidence1, "% probability that the number of [primary outcome events] will be between ", "<u>",input$round1range1[1]," and ", input$round1range1[2],"</u>", ",

and ","<u>", input$round1estimate1,"</u>", " patients will mostly likely experience [primary outcome].","</b>")) # Modify

})

output$summaryr1q1<-renderUI(r1q1text())

essr1q1text <- reactive({

round1q1_lower<-adjust_value(input$round1range1[1]/20)

round1q1_upper<-adjust_value(input$round1range1[2]/20)

round1q1_estimate<-adjust_value(input$round1estimate1/20)

round1q1_betaparam<-betaExpert(round1q1_estimate,round1q1_lower,round1q1_upper,p=input$round1confidence1/100,

method="mode")

round1q1_a<-round1q1_betaparam$alpha

round1q1_b<-round1q1_betaparam$beta

round1q1_ess <- round(round1q1_a+round1q1_b, 2)

HTML(paste0("Your effective sample size (ESS) is ", "<b><u>", round1q1_ess,

"</b></u>."))

})

output$essr1q1 <- renderUI(essr1q1text())

\# Round1 Question1 Plot

r1q1plot<-reactive({

round1q1_lower<-adjust_value(input$round1range1[1]/20)

round1q1_upper<-adjust_value(input$round1range1[2]/20)

round1q1_estimate<-adjust_value(input$round1estimate1/20)

round1q1_betaparam<-betaExpert(round1q1_estimate,round1q1_lower,round1q1_upper,p=input$round1confidence1/100,

method="mode")

round1q1_a<-round1q1_betaparam$alpha

round1q1_b<-round1q1_betaparam$beta

round1q1_p<-seq(0,1,length=100)

r1q1data<-data.frame(x=round1q1_p,y=dbeta(round1q1_p,round1q1_a,round1q1_b))

ggplot(data=r1q1data,aes(x,y))+geom_line(color="black",size=1)+

labs(title = "Probability distribution for ...",x="...",y="")+ # Modify

scale_x_continuous(breaks=c(0,0.2,0.4,0.6,0.8,1),labels=c(0,0.2,0.4,0.6,0.8,1))+

theme(plot.title = element_text(size=14, hjust = 0.5))+

theme(panel.border = element_blank())+

theme(axis.text.x = element_text(size=12,face="bold"))+

theme(axis.line.x = element_line(size=1,colour="black"))+

theme(axis.title.x = element_text(size=14))+

theme(axis.ticks.y=element_blank(),axis.text.y = element_blank())+

geom_area(data=subset(r1q1data,x>=qbeta(0.025,round1q1_a,round1q1_b)& x<=qbeta(0.975,round1q1_a,round1q1_b)),aes(x=x,y=y),colour="black",fill="lightblue",alpha=1)

})

output$plotr1q1<-renderPlot(r1q1plot())

\# Round 1 Question 2 slider input update

observe({

updateSliderInput(session = session, "round1estimate2", value = round((input$round1range2[1]+input$round1range2[2])/2,0), min = input$round1range2[1],

max=input$round1range2[2])})

\# Round 1 Question 2 Summary

r1q2text<-reactive({

round1q2_lower<-input$round1range2[1]/20

round1q2_upper<-input$round1range2[2]/20

round1q2_estimate<-input$round1estimate2/20

HTML(paste0("Based on your input, out of 20 similiar patients with [disease] and treated with [intervention]

,","<b>", " there is ", input$round1confidence2, "% probability that the number of [primary outcome events] will be between ", "<u>",input$round1range2[1]," and ", input$round1range2[2],"</u>", ",

and ","<u>", input$round1estimate2,"</u>", " patients will mostly likely experience [primary outcome].","</b>")) # Modify

})

output$summaryr1q2<-renderUI(r1q2text())

essr1q2text <- reactive({

round1q2_lower<-adjust_value(input$round1range2[1]/20)

round1q2_upper<-adjust_value(input$round1range2[2]/20)

round1q2_estimate<-adjust_value(input$round1estimate2/20)

round1q2_betaparam<-betaExpert(round1q2_estimate,round1q2_lower,round1q2_upper,p=input$round1confidence2/100,

method="mode")

round1q2_a<-round1q2_betaparam$alpha

round1q2_b<-round1q2_betaparam$beta

round1q2_ess <- round(round1q2_a+round1q2_b, 2)

HTML(paste0("Your effective sample size (ESS) is ", "<b><u>", round1q2_ess,

"</b></u>."))

})

output$essr1q2 <- renderUI(essr1q2text())

\# Round1 Question2 Plot

r1q2plot<-reactive({

round1q2_lower<-adjust_value(input$round1range2[1]/20)

round1q2_upper<-adjust_value(input$round1range2[2]/20)

round1q2_estimate<-adjust_value(input$round1estimate2/20)

round1q2_betaparam<-betaExpert(round1q2_estimate,round1q2_lower,round1q2_upper,p=input$round1confidence2/100,

method="mode")

round1q2_a<-round1q2_betaparam$alpha

round1q2_b<-round1q2_betaparam$beta

round1q2_p<-seq(0,1,length=100)

r1q2data<-data.frame(x=round1q2_p,y=dbeta(round1q2_p,round1q2_a,round1q2_b))

ggplot(data=r1q2data,aes(x,y))+geom_line(color="black",size=1)+

labs(title = "Probability distribution for ...",x="...",y="")+ # Modify

scale_x_continuous(breaks=c(0,0.2,0.4,0.6,0.8,1),labels=c(0,0.2,0.4,0.6,0.8,1))+

theme(plot.title = element_text(size=14, hjust = 0.5))+

theme(panel.border = element_blank())+

theme(axis.text.x = element_text(size=12,face="bold"))+

theme(axis.line.x = element_line(size=1,colour="black"))+

theme(axis.title.x = element_text(size=14))+

theme(axis.ticks.y=element_blank(),axis.text.y = element_blank())+

geom_area(data=subset(r1q2data,x>=qbeta(0.025,round1q2_a,round1q2_b)& x<=qbeta(0.975,round1q2_a,round1q2_b)),aes(x=x,y=y),colour="black",fill="lightblue",alpha=1)

})

output$plotr1q2<-renderPlot(r1q2plot())

\# Group Discussion ________________________________________________________________________________________________________________________

discussionred1<-reactive(

HTML(paste("For this question, your lower plausible value is","<b>",input$round1range1[1],"</b>"," and the upper

plausible value is","<b>",input$round1range1[2],"</b>",". The best/most likely

value is","<b>",input$round1estimate1,"</b>",

". Your confidence level is", "<b>", input$round1confidence1, "% </b>.")))

output$discussionredcap1<-renderUI(discussionred1())

output$discussiontext1<-renderUI(r1q1text())

output$discussioness1 <- renderUI(essr1q1text())

output$discussionplot1<-renderPlot(r1q1plot())

discussionred2<-reactive(

HTML(paste("For this question, your lower plausible value is","<b>",input$round1range2[1],"</b>"," and the upper

plausible value is","<b>",input$round1range2[2],"</b>",". The best/most likely

value is","<b>",input$round1estimate2,"</b>",

". Your confidence level is", "<b>", input$round1confidence2, "% </b>.")))

output$discussionredcap2<-renderUI(discussionred2())

output$discussiontext2<-renderUI(r1q2text())

output$discussioness2 <- renderUI(essr1q2text())

output$discussionplot2<-renderPlot(r1q2plot())

\# Round 2 Question 1 slider input update

observe({

updateSliderInput(session = session, "round2estimate1", value = round((input$round2range1[1]+input$round2range1[2])/2,0), min = input$round2range1[1],

max=input$round2range1[2])})

\# Round 2 Question 1 Summary

r2q1text<-reactive({

round2q1_lower<-input$round2range1[1]/20

round2q1_upper<-input$round2range1[2]/20

round2q1_estimate<-input$round2estimate1/20

HTML(paste0("Based on your input, out of 20 similiar patients with [disease] and treated with [comparator]

,","<b>", " there is ", input$round2confidence1, "% probability that the number of [primary outcome events] will be between ", "<u>",input$round2range1[1]," and ", input$round2range1[2],"</u>", ",

and ","<u>", input$round2estimate1,"</u>", " patients will mostly likely experience [primary outcome].","</b>")) # Modify

})

output$summaryr2q1<-renderUI(r2q1text())

essr2q1text <- reactive({

round2q1_lower<-adjust_value(input$round2range1[1]/20)

round2q1_upper<-adjust_value(input$round2range1[2]/20)

round2q1_estimate<-adjust_value(input$round2estimate1/20)

round2q1_betaparam<-betaExpert(round2q1_estimate,round2q1_lower,round2q1_upper,p=input$round2confidence1/100,

method="mode")

round2q1_a<-round2q1_betaparam$alpha

round2q1_b<-round2q1_betaparam$beta

round2q1_ess <- round(round2q1_a+round2q1_b, 2)

HTML(paste0("Your effective sample size (ESS) is ", "<b><u>", round2q1_ess,

"</b></u>."))

})

output$essr2q1 <- renderUI(essr2q1text())

\# Round2 Question1 Plot

r2q1plot<-reactive({

round2q1_lower<-adjust_value(input$round2range1[1]/20)

round2q1_upper<-adjust_value(input$round2range1[2]/20)

round2q1_estimate<-adjust_value(input$round2estimate1/20)

round2q1_betaparam<-betaExpert(round2q1_estimate,round2q1_lower,round2q1_upper,p=input$round2confidence1/100,

method="mode")

round2q1_a<-round2q1_betaparam$alpha

round2q1_b<-round2q1_betaparam$beta

round2q1_p<-seq(0,1,length=100)

r2q1data<-data.frame(x=round2q1_p,y=dbeta(round2q1_p,round2q1_a,round2q1_b))

ggplot(data=r2q1data,aes(x,y))+geom_line(color="black",size=1)+

labs(title = "Probability distribution for ...",x="...",y="")+ # Modify

scale_x_continuous(breaks=c(0,0.2,0.4,0.6,0.8,1),labels=c(0,0.2,0.4,0.6,0.8,1))+

theme(plot.title = element_text(size=14, hjust = 0.5))+

theme(panel.border = element_blank())+

theme(axis.text.x = element_text(size=12,face="bold"))+

theme(axis.line.x = element_line(size=1,colour="black"))+

theme(axis.title.x = element_text(size=14))+

theme(axis.ticks.y=element_blank(),axis.text.y = element_blank())+

geom_area(data=subset(r2q1data,x>=qbeta(0.025,round2q1_a,round2q1_b)& x<=qbeta(0.975,round2q1_a,round2q1_b)),aes(x=x,y=y),colour="black",fill="lightblue",alpha=1)

})

output$plotr2q1<-renderPlot(r2q1plot())

\# Round 2 Question 2 slider input update

observe({

updateSliderInput(session = session, "round2estimate2", value = round((input$round2range2[1]+input$round2range2[2])/2,0), min = input$round2range2[1],

max=input$round2range2[2])})

\# Round 2 Question 2 Summary

r2q2text<-reactive({

round2q2_lower<-input$round2range2[1]/20

round2q2_upper<-input$round2range2[2]/20

round2q2_estimate<-input$round2estimate2/20

HTML(paste0("Based on your input, out of 20 similiar patients with [disease] and treated with [intervention]

,","<b>", " there is ", input$round2confidence2, "% probability that the number of [primary outcome events] will be between ", "<u>",input$round2range2[1]," and ", input$round2range2[2],"</u>", ",

and ","<u>", input$round2estimate2,"</u>", " patients will mostly likely experience [primary outcome].","</b>")) # Modify

})

output$summaryr2q2<-renderUI(r2q2text())

essr2q2text <- reactive({

round2q2_lower<-adjust_value(input$round2range2[1]/20)

round2q2_upper<-adjust_value(input$round2range2[2]/20)

round2q2_estimate<-adjust_value(input$round2estimate2/20)

round2q2_betaparam<-betaExpert(round2q2_estimate,round2q2_lower,round2q2_upper,p=input$round2confidence2/100,

method="mode")

round2q2_a<-round2q2_betaparam$alpha

round2q2_b<-round2q2_betaparam$beta

round2q2_ess <- round(round2q2_a+round2q2_b, 2)

HTML(paste0("Your effective sample size (ESS) is ", "<b><u>", round2q2_ess,

"</b></u>."))

})

output$essr2q2 <- renderUI(essr2q2text())

\# Round2 Question2 Plot

r2q2plot<-reactive({

round2q2_lower<-adjust_value(input$round2range2[1]/20)

round2q2_upper<-adjust_value(input$round2range2[2]/20)

round2q2_estimate<-adjust_value(input$round2estimate2/20)

round2q2_betaparam<-betaExpert(round2q2_estimate,round2q2_lower,round2q2_upper,p=input$round2confidence2/100,

method="mode")

round2q2_a<-round2q2_betaparam$alpha

round2q2_b<-round2q2_betaparam$beta

round2q2_p<-seq(0,1,length=100)

r2q2data<-data.frame(x=round2q2_p,y=dbeta(round2q2_p,round2q2_a,round2q2_b))

ggplot(data=r2q2data,aes(x,y))+geom_line(color="black",size=1)+

labs(title = "Probability distribution for ...",x="...",y="")+ # Modify

scale_x_continuous(breaks=c(0,0.2,0.4,0.6,0.8,1),labels=c(0,0.2,0.4,0.6,0.8,1))+

theme(plot.title = element_text(size=14, hjust = 0.5))+

theme(panel.border = element_blank())+

theme(axis.text.x = element_text(size=12,face="bold"))+

theme(axis.line.x = element_line(size=1,colour="black"))+

theme(axis.title.x = element_text(size=14))+

theme(axis.ticks.y=element_blank(),axis.text.y = element_blank())+

geom_area(data=subset(r2q2data,x>=qbeta(0.025,round2q2_a,round2q2_b)& x<=qbeta(0.975,round2q2_a,round2q2_b)),aes(x=x,y=y),colour="black",fill="lightblue",alpha=1)

})

output$plotr2q2<-renderPlot(r2q2plot())

}

shinyApp(ui, server)
