# Supplementary material for: A primer on expert elicitation with IDEA (Investigate Discuss Estimate Aggregate) for rare disease trialists
Source: J Rare Dis (Berlin). 2026 Nov 4;5(1):111. doi: 10.1007/s44162-026-00253-8 (PMC13736339; doi:10.1007/s44162-026-00253-8)
Supplement: Supplementary file 4 [file 44162_2026_253_MOESM4_ESM.docx]

\# The following code performs equal-weighted linear pooling for a binary

\# primary outcome. Please modify with data from your elicitation.

library(prevalence)

\# Second Round DATA

k <- 10 # Modify with number of experts

\# Modify with elicitation question 1 answers

dataset1 <- data.frame(

best = rep(.10, 10),

lower = rep(.01, 10),

upper= rep(.15, 10),

conf = rep(.9, 10)

)

\# Modify with elicitation question 2 answers

dataset2 <- data.frame(

best = rep(.10, 10),

lower = rep(.01, 10),

upper= rep(.15, 10),

conf = rep(.9, 10)

)

\# Parameter Estimation

alpha1<-c()

beta1<-c()

for (i in 1:k){

alpha1[i]<-betaExpert(dataset1$best[i],dataset1$lower[i],dataset1$upper[i],p=dataset1$conf[i], method = "mode")$alpha

beta1[i]<-betaExpert(dataset1$best[i],dataset1$lower[i],dataset1$upper[i],p=dataset1$conf[i],method = "mode")$beta

}

alpha2<-c()

beta2<-c()

for (i in 1:k){

alpha2[i]<-betaExpert(dataset2$best[i],dataset2$lower[i],dataset2$upper[i],p=dataset2$conf[i], method = "mode")$alpha

beta2[i]<-betaExpert(dataset2$best[i],dataset2$lower[i],dataset2$upper[i],p=dataset2$conf[i], method = "mode")$beta

}

\# Linear Pool

x_vals <- seq(0, 1, length.out = 200)

fx_matrix1 <- matrix(NA, nrow = 200, ncol = k)

for (i in 1:k) {

fx_matrix1[, i] <- dbeta(x_vals, alpha1[i], beta1[i])

}

fx_pooled1 <- rowMeans(fx_matrix1)

plot(x_vals, fx_pooled1)

fx_matrix2 <- matrix(NA, nrow = 200, ncol = k)

for (i in 1:k) {

fx_matrix2[, i] <- dbeta(x_vals, alpha2[i], beta2[i])

}

fx_pooled2 <- rowMeans(fx_matrix2)

plot(x_vals, fx_pooled2)
