# Supplementary material for: A primer on expert elicitation with IDEA (Investigate Discuss Estimate Aggregate) for rare disease trialists
Source: J Rare Dis (Berlin). 2026 Nov 4;5(1):111. doi: 10.1007/s44162-026-00253-8 (PMC13736339; doi:10.1007/s44162-026-00253-8)
Supplement: Supplementary file 5 [file 44162_2026_253_MOESM5_ESM.docx]

Please adopt this template to fit your study and ethics approval process. Feel free to reuse any figures and text supplied here.

EXPERT ELICITATION OF PRIOR PROBABILITY DISTRIBUTION FOR A RANDOMIZED CONTROLLED TRIAL COMPARING [INTERVENTION] TO [COMPARATOR] TO TREAT [POPULATION OF INTEREST]

| Principal Investigator: |  |
| --- | --- |
| Sponsor: |  |
| Funding by: |  |
| Version Date [Day, Month, Year] |  |

**Table of Contents**

[1 PROTOCOL SUMMARY	1](#_Toc201310613)

[1.1 Synopsis	1](#_Toc201310614)

[1.2 Schematic of Study Design	3](#_Toc201310615)

[1.3 Schedule of Activities (SOA)	4](#_Toc201310616)

[1.4 Key Roles	4](#_Toc201310617)

[2 Introduction, Background Information and Scientific Rationale	5](#_Toc201310618)

[2.1 Background Information and Relevant Literature	5](#_Toc201310619)

[2.2	Rationale	5](#_Toc201310620)

[2.3	Potential Risks & Benefits	5](#_Toc201310621)

[2.3.1 Potential Risks	5](#_Toc201310622)

[2.3.2 Potential Benefits	6](#_Toc201310623)

[3 Objectives and Purpose	6](#_Toc201310624)

[3.1	Primary Objective	6](#_Toc201310625)

[4	Study Design and Endpoints	6](#_Toc201310626)

[4.1 Description of Study Design	6](#_Toc201310627)

[4.2	Duration of Study Participation	7](#_Toc201310628)

[4.3	Total Number of Participants and Sites	7](#_Toc201310629)

[5	Study Enrollment and Withdrawal	7](#_Toc201310630)

[5.1 Participant Inclusion Criteria	7](#_Toc201310631)

[5.2	Participant Exclusion Criteria	7](#_Toc201310632)

[5.3	Strategies for Recruitment and Retention	8](#_Toc201310633)

[5.4	Participant Withdrawal or Termination	8](#_Toc201310634)

[5.4.1 Reasons for Withdrawal or Termination	8](#_Toc201310635)

[5.4.2 Handling of Participant Withdrawals or Termination	9](#_Toc201310636)

[6 Study Assessments and Procedures	9](#_Toc201310637)

[7 Study Procedures / Evaluations	9](#_Toc201310638)

[7.1	Procedures / Evaluations	9](#_Toc201310639)

[7.2	Questionnaire Administration	10](#_Toc201310640)

[8 Statistical Considerations	10](#_Toc201310641)

[8.1 Sample Size Determination for Elicitation Process	10](#_Toc201310642)

[8.2 Statistical Analysis to Obtain Prior Probability Distribution	11](#_Toc201310643)

[To formulate the expert’s beliefs into the prior probability distribution, we assume the prior probability distribution will follow the beta distribution, which is bounded by [0,1].	11](#_Toc201310644)

[8.3 Sample Size Determination for the BIPED Study	11](#_Toc201310645)

[9 Source Documents and Access to Source Data / Documents	11](#_Toc201310646)

[10 Study Oversight	12](#_Toc201310647)

[11 Ethics / Protection of Human Participants	12](#_Toc201310648)

[11.1	Ethical Standard	12](#_Toc201310649)

[11.2	Informed Consent Process and Documentation	12](#_Toc201310650)

[11.3	Consent / Assent and Other Informational Documents Provided to Participants	12](#_Toc201310651)

[12 Data Handling and Record Keeping	13](#_Toc201310652)

[12.1 Data Collection and Management Responsibilities	13](#_Toc201310653)

[12.2 Study Records Retention	13](#_Toc201310654)

[13 Protocol Deviations	13](#_Toc201310655)

[14 Publication and Data Sharing Policy	13](#_Toc201310656)

[15 References	14](#_Toc201310657)

List of Abbreviations

| PI | Principal Investigator |
| --- | --- |
| REB | Research Ethics Board |
| SSD | Sample Size Determination |
| SOA | Schedule of Activities |

# 1 PROTOCOL SUMMARY

## 1.1 Synopsis

| Title | Expert elicitation of prior probability distribution for a randomized controlled trial comparing [intervention] to [comparator] in [population of interest] |
| --- | --- |
| Short Title | Elicitation for [trial title] |
| Study Description | We will conduct an expert elicitation process to obtain expert opinion, together with uncertainty, regarding [clinical question]. |
| Objectives | The primary objective of this study is to elicit and formulate experts’ judgements into an informative prior distribution for [trial title]. |
| Study Design | A prospective study using a virtual workshop and questionnaire to elicit expert judgements on [clinical question] |
| Participant Population, Selection Criteria | We will enroll [number of experts] experts across [regions for experts]. We will enroll participants who are identified as experts in [disease] and its treatment by the study investigators. Experts must be professionals with experience in [disease area]. |
| Study Sites | [your research center] |
| Participant Duration | Participants will be followed (by email) for 30 days after enrolment |
| Number of participants | [number of experts] participates |
| Study Phases (Screening, Study Treatment, Follow-Up) | Not applicable |
| Efficacy Evaluations | The primary outcome for this study is the expert beliefs on [elicitation quantities] |
| Safety Evaluations | Not applicable |
| Statistical Analysis | An equal-weighted linear pool [/alternative pooling method] is used to aggregate individual probability distributions to form a prior probability distribution. |
| Data and Safety Monitoring Plan | Principal Investigator is responsible for all data quality management and privacy |

## 1.2 Schematic of Study Design

N=X: recruiting potential participants by inclusion and exclusion criteria; obtain informed consent.

Prior to

Enrollment

Allocate to Workshops

Pre-elicitation

Emailed the study background, evidence dossier

and elicitation process to experts

Day 0

Elicitation: First round (Workshop)

Experts’ judgements for the quantities of interest collected

Day 7

(+7) days

Elicitation: Group Discussion (Workshop)

Experts discussion about first round judgements

Elicitation: Second round (Workshop)

Experts’ judgements for the quantities of interest collected

(Same questions as first round)

Post- Elicitation: Follow-up Assessments

Email the results to experts to adjust the aggregated prior probability distribution

Day 14

(+7) days

Final Assessments:

individual distribution and aggregated distribution review

Day 21

## 1.3 Schedule of Activities (SOA)

| Procedures | Prior Enrolment | Enrolment  (Day 0) | Workshop  (Day 7) | Feedback  (Day 14) | Day 21 |
| --- | --- | --- | --- | --- | --- |
| Time Window |  |  | +7 days | +7 days |  |
| Inclusion criteria | X |  |  |  |  |
| Informed consent | X |  |  |  |  |
| Demographics | X |  | X |  |  |
| Workshop preparation |  | X |  |  |  |
| Zoom workshop |  |  | X |  |  |
| Email follow-up |  |  |  | X |  |
| Plot Review/ Data Review |  |  |  |  | X |

## 1.4 Key Roles

Principal Investigator for the Elicitation Study

[contact for Principal Investigator for the Elicitation Study]

Analyst for Elicitation Study

[contact for Analyst for the Elicitation Study]

Facilitator for Elicitation Study

[contact for Facilitator for the Elicitation Study]

Coordinator for Elicitation Study

[contact for Coordinator for the Elicitation Study]

Sponsor Investigator for [trial name]

[contact for Sponsor Investigator]

# 2 Introduction, Background Information and Scientific Rationale

## 2.1 Background Information and Relevant Literature

[Insert background information about the relevance of your clinical research question]

Members of our team have proposed a clinical trial that will evaluate [clinical question]. The study will use a Bayesian design and analysis, which allows for the incorporation of prior information. This prior information can be obtained from earlier studies, or in their absence, from expert knowledge.^1^

Expert elicitation techniques have been increasingly applied to inform decision making when data are insufficient.^2-4^ It can be used to inform priors for Bayesian statistical model.^1^ Examples include treatment for trauma resuscitation,^5^ risk factors in salmon farming,^4^ negative pressure wound therapy and photo acoustic mammography.^6^ In a structured expert elicitation process, experts will be assisted in converting their knowledge into a quantitative expression, with each step viewed as a formal data acquisition based on validated methodologies.^4^

This study aims to conduct a structured expert elicitation process to elicit current beliefs about the [elicitation quantities]. The elicited prior distribution will then be used for the design and analysis of the [clinical trial]. The [clinical trial] has received ethical approval from all study sites in accordance with local research ethics board guidelines. This protocol exclusively discusses the proposed expert elicitation process.

## 2.2 Rationale

One of the benefits of using Bayesian statistical models is to combine observed data with other “prior” information when the observed data are limited in sample size or representativeness^1^. The prior information can be obtained from expert knowledge through an elicitation process. In this study, we will enrol experts to transform their knowledge in the treatment of [disease] into quantitative expressions. These expressions will then be used as the prior information in the design and analysis of the [clinical trial].

## 2.3 Potential Risks & Benefits

### 2.3.1 Potential Risks

This study aims to elicit knowledge from experts who have expertise in [disease]. The only potential risks to experts in this study is that they can potentially be identified from their elicitation data.

### 2.3.2 Potential Benefits

This study is a key part of designing [trial title], which will generate evidence to benefit patients with [disease].

# 3 Objectives and Purpose

The primary objective of this study is to elicit and formulate experts’ judgements into an informative prior distribution for [trial title]. Expert elicitation will be used to obtain expert judgements with uncertainty. These expert judgements are formulated into an informative prior distribution for a Bayesian clinical trial.

## 3.1 Primary Objective

| Objective | Brief Description / Justification of Outcome Measure | Outcome Measured By | Time Frame |
| --- | --- | --- | --- |
|  |  |  |  |

# 4 Study Design and Endpoints

## 4.1 Description of Study Design

Members of our team have proposed a clinical trial that will evaluate [trial research question]. To determine the prior distribution for this Bayesian clinical trial, this study will focus on eliciting judgements from experts. This study will conduct a [time frame] online expert elicitation process. [Number of experts] experts with expertise in [disease] will participate in an online workshop, where we will collect their judgements on [research question]. Their judgement will be converted to an individual probability distribution. We will then use equal-weighted linear pooling of the individual distributions to calculate the prior distribution for [trial title].^5^

## 4.2 Duration of Study Participation

The study enrolment will start in [start date]. [Number of workshops] workshops will be held to accommodate expert availability. After the workshops, the facilitator will send the summary of aggregated expert judgements across workshops to participants in 5-7 days. The facilitator will collect feedback and comments on the results and include them in the final report. The final prior probability distribution will be used in the design of the trial.

## 4.3 Total Number of Participants and Sites

Overall, we aim to recruit 10-20 participants into the study, as recommended by the IDEA protocol.^7^ The elicitation process will take place through [elicitation platform] hosted at the [research institute].

# 5 Study Enrollment and Withdrawal

## 5.1 Participant Inclusion Criteria

In line with other elicitation process,^4,5^ to be eligible to participate in this study, an individual must meet all of the following criteria:

- Individuals who are identified as experts in [disease] and its treatment by the trial investigators.
- Professionals with experience in the [disease area]
- [Add any relevant inclusion/exclusion criteria]

## 5.2 Participant Exclusion Criteria

An individual who meets any of the following criteria will be excluded from participation in this study:

- Individuals with prior involvement in the [trial title]

## 5.3 Strategies for Recruitment and Retention

As this study requires the involvement of experts in [disease], we will be recruiting professionals (i.e., researchers, clinicians, policy makers) from around the world. These professionals will be identified by the trial investigators through their professional networks. Consent to take part in the study will be obtained by email.

Participants will be provided with the study materials and invited to a virtual workshop. We will send calendar invitations to the workshop and send one reminder email 24 hours before the workshop is due to take place. Following the workshop, participants who attend will be sent the aggregated distribution. They will be asked to provide their feedback on the distribution. Participants who do not respond with their updated distributions will be followed up by email three times. Participants who do not respond with their updated distributions will still be included in the final analysis. Participants who do not attend the virtual workshop will be excluded.

## 5.4 Participant Withdrawal or Termination

### 5.4.1 Reasons for Withdrawal or Termination

Participants are free to withdraw from taking part in the study at any time upon request. An investigator may terminate participation in the study if:

- This elicitation study is temporarily suspended or prematurely terminated.
- The participant meets an exclusion criterion (either newly developed or not previously recognized) that precludes further study participation.

### 5.4.2 Handling of Participant Withdrawals or Termination

The reason for participant withdrawal from the study will be recorded. If participants complete the workshop without providing feedback, they will not be replaced and their outcomes will be included in the final analysis. Subjects who indicate they will take part in the elicitation exercise but do not complete the workshop will be not included in the analysis.

# 6 Study Assessments and Procedures

Once the trial investigators have received provisional consent from participants via email, the elicitation facilitator will reach out to these participants with a consent form and participation information form.

# 7 Study Procedures / Evaluations

## 7.1 Procedures / Evaluations

**Pre-Elicitation:** The research team will identify the quantities of interest and develop elicitation questions, such as [insert elicitation question]. One week before the formal elicitation, the facilitator will send the elicitation process information to the participants.

**Elicitation:** During the elicitation, participants will have two opportunities to answer the elicitation questions. [Describe the process of using your elicitation tool]. In addition, the participants will have opportunity to discuss their judgements after the first-round questionnaire. The purpose of the group discussion is to help experts calibrate their opinion instead of coming to a consensus^5^. After the group discussion, the participants can revise their views in the second-round questionnaire.

**Post-Elicitation:** Participants will be emailed at day 5 or 7 following the workshop. The email will consist of the statements and summaries about the aggregated fitted distributions across two workshops in a similar format to the original questions. This email will seek feedback on whether the fitted distribution represents the participant’s beliefs. If there is no feedback, the facilitator will send the email at most three times in a row. In the end, the facilitator will collect feedback and comments on the results and include them in the final report.

All the data will be analyzed at a password protected [research institute] workstation.

## 7.2 Questionnaire Administration

Elicitation workshops will be conducted through Zoom meetings. Under the facilitator’s guide, the expert will complete and submit their answers to a questionnaire. This questionnaire has been previously reviewed and validated by the study investigator and two external experts.

# 8 Statistical Considerations

## 8.1 Sample Size Determination for Elicitation Process

We chose to include 10 to 20 experts, as recommended by the IDEA protocol.^7^ This number of participants will allow us to capture a diversity of expert opinions, while maintaining study feasibility.

## 8.2 Statistical Analysis to Obtain Prior Probability Distribution

To formulate the expert’s beliefs into the prior probability distribution, we assume the prior probability distribution will follow the [insert appropriate distribution] distribution.

To obtain the prior probability, we will first convert expert judgements into several [insert appropriate distribution] distributions.To aggregate the individual elicited distribution, an equal-weighted linear pooling [/insert alternative pooling method] will be used. This method can help to derive group prior with the extensive overlap of all the individual priors. It also helps to mitigate overconfidence and overoptimism.^5,8^

In the end, we will derive aggregated distributions, representing …

# 9 Source Documents and Access to Source Data / Documents

Access to study records will be limited to REB-approved members of the study team. The PI will permit study-related monitoring, audits, and inspections by the REB and / or University compliance and quality assurance groups of all study related documents (e.g. source documents, data collection instruments, study data etc.).

Participation as a PI in this study implies acceptance of potential inspection by REB and / or applicable University compliance and quality assurance offices.

# 10 Study Oversight

The [research institute] Research Ethics Board will review this study and be responsible for monitoring. The Study PI will oversee the project and ensure it conforms, where required, to the regulations and guidelines of the [research institute].

# 11 Ethics / Protection of Human Participants

## 11.1 Ethical Standard

We will conduct this study in accordance with the Tri-Council Policy Statement: Ethical Conduct for Research Involving Humans (2022) and the procedures set out by the [research institute] Research Ethics Board.

## 11.2 Informed Consent Process and Documentation

Consent forms will be REB-approved, and the participant will be asked to read and review the document. Research personnel will provide an information dossier via email to the participant and answer any questions that may arise. If requested, study personnel will be available to offer a verbal explanation of the study to aid the participant’s comprehension of the purposes, procedures, and their rights as research participants. Participants will have the opportunity to carefully review the written consent form and ask questions before signing. Participants will be informed that participation is voluntary and that they may withdraw from the study at any time. A copy of the informed consent document will be given to the participant for their records. The informed consent process will be documented using a study specific checklist.

## 11.3 Consent / Assent and Other Informational Documents Provided to Participants

Consent forms describing in detail the study procedures will be given to the participant and written documentation of informed consent is required before starting the elicitation process.

# 12 Data Handling and Record Keeping

## 12.1 Data Collection and Management Responsibilities

Data collection is the responsibility of the study staff at the site under the supervision of the site PI. The PI is responsible for ensuring the accuracy, completeness, legibility, and timeliness of the data reported.

[Please specify your data management plan].

## 12.2 Study Records Retention

The study data will be stored at the [research institute] until all data management and statistical analysis activity has been completed. The data will then be stored for 25 years to match the data storage requirements for data from clinical trials^9^.

# 13 Protocol Deviations

The protocol deviations in this study will be reviewed once all the results have been collected. Provided the participant attended the virtual workshop, their data will be included in the final analysis. Any participant who did not attend the workshop will be excluded.

# 14 Publication and Data Sharing Policy

All team members listed under Key Roles (section 1.4) will be included as an author on the publication of the elicitation. Data will not be shared externally to [research institute] but the results of the elicitation exercise will be shared with other study investigators and through publication.

# 15 References

1. Choy, S. L., O'Leary, R., & Mengersen, K. (2009). Elicitation by design in ecology: using expert opinion to inform priors for Bayesian statistical models. Ecology, 90(1), 265-277.
2. Martin, T. G., Burgman, M. A., Fidler, F., Kuhnert, P. M., Low‐Choy, S. A. M. A. N. T. H. A., McBride, M., & Mengersen, K. (2012). Eliciting expert knowledge in conservation science. Conservation Biology, 26(1), 29-38.
3. Mellers, B., Ungar, L., Baron, J., Ramos, J., Gurcay, B., Fincher, K., ... & Murray, T. (2014). Psychological strategies for winning a geopolitical forecasting tournament. Psychological science, 25(5), 1106-1115.
4. Estévez, R. A., Mardones, F. O., Álamos, F., Arriagada, G., Carey, J., Correa, C., ... & Ortiz, C. (2019). Eliciting expert judgements to estimate risk and protective factors for Piscirickettsiosis in Chilean salmon farming. Aquaculture, 507, 402-410.
5. Jansen, J. O., Wang, H., Holcomb, J. B., Harvin, J. A., Richman, J., Avritscher, E., ... & Yamal, J. M. (2020). Elicitation of prior probability distributions for a proposed Bayesian randomized clinical trial of whole blood for trauma resuscitation. Transfusion, 60(3), 498-506.
6. Dias, L. C., Morton, A., & Quigley, J. (2018). Elicitation. Springer International Publishing. MR3700912. doi: https://doi. org/10.1007/978-3-319-65052-4, 1(2), 3.
7. Hemming V, Burgman MA, Hanea AM, McBride MF, Wintle BC. A practical guide to structured expert elicitation using the IDEA protocol. Methods Ecol Evol. 2017 Jul 30;9(1):169–80.
8. Johnson, Sindhu R., et al. "Methods to elicit beliefs for Bayesian priors: a systematic review." Journal of clinical epidemiology 63.4 (2010): 355-369.
9. Guidance for Records Related to Clinical Trials (Guide-0068) -Interpretation of section C.05.012 of the Food and Drug Regulations – Division 5 “Drugs for clinical trials involving human subjects” – Health Canada, [Accessed: 02/12/2020], available at: <https://www.queensu.ca/urs/sites/webpublish.queensu.ca.urwww/files/files/HSREB/Health%20Canada%20Guidance%20for%20Records%20Related%20to%20Clincial%20trials%20Guide%200068.pdf>
