# Supplementary material for: A primer on expert elicitation with IDEA (Investigate Discuss Estimate Aggregate) for rare disease trialists
Source: J Rare Dis (Berlin). 2026 Nov 4;5(1):111. doi: 10.1007/s44162-026-00253-8 (PMC13736339; doi:10.1007/s44162-026-00253-8)
Supplement: Supplementary file 6 [file 44162_2026_253_MOESM6_ESM.docx]

The following 3 files are adapted from Thall et al. (2017). The first file is an R Markdown file. The second two files are stan files.

R Markdown file begins:

---

title: 'Thall et al. (2017) Aggregation'

output:

html_document:

df_print: paged

---

The following R Markdown code was adapted from the code in the Thall et al. (2017).

\## Step 1. Import libraries and data

Modify with your data.

```{r}

rm(list=ls())

library(openxlsx)

library(prevalence)

library(rstan)

library(mvtnorm)

library(parallel)

library(bayesplot)

library(grr)

ncores <- detectCores()-2

q1_data <- # Modify

q2_data <- # Modify

covariates <- # Modify

names(covariates) <- c("EXPERT.ID", "Q1", "Q2", "Q3", "Q4", "Q5", "Q6", "Q7") # Modify

\# Sort dataframes by expert ID

q1_data <- q1_data[order(q1_data$EXPERT.ID),]

q2_data <- q2_data[order(q2_data$EXPERT.ID),]

covariates <- covariates[order(covariates$EXPERT.ID),]

\# Extract relevant values

K <- dim(covariates)[1] # Number of experts

B <- dim(covariates)[2] - 1 # Number of covariates

```

\## Step 2. Clean covariate data

```{r}

\# Clean numeric data - [-1] to remove expert indices

numInd <- unname(which(sapply(covariates, is.numeric)))[-1]

\# Check that log-transform improves normality

par(mfrow=c(1,2))

for (i in numInd) {

hist((covariates[,i]), breaks=8, main=names(covariates[i]), xlab='')

hist((log(covariates[,i])), breaks=8,

main=paste(names(covariates[i]), "Log-Transformed"), xlab='')

}

\# Log-transform variables

covariates[,numInd] <- log(covariates[,numInd])

\# Clean other variables

catInd <- unname(which(sapply(covariates, is.character)))

covariates[,catInd] <- ifelse(covariates[,catInd]=="Yes", 1, 0)

```

\## Step 3. Fit beta distributions

```{r}

\# Probability

q1_data[,-1] <- q1_data[,-1] / 100

q2_data[,-1] <- q2_data[,-1] / 100

alpha1 <- rep(NA, K)

beta1 <- rep(NA, K)

alpha2 <- rep(NA, K)

beta2 <- rep(NA, K)

for (i in 1:K){

b <- betaExpert(q1_data[i,4], q1_data[i,2], q1_data[i,3])

alpha1[i] <- b$alpha

beta1[i] <- b$beta

b <- betaExpert(q2_data[i,4], q2_data[i,2], q2_data[i,3])

alpha2[i] <- b$alpha

beta2[i] <- b$beta

}

```

\## Step 4. Calculate mean and precision for each distribution.

```{r}

mu1 <- alpha1/(alpha1 + beta1)

gamma1 <- alpha1 + beta1

mu2 <- alpha2/(alpha2 + beta2)

gamma2 <- alpha2 + beta2

```

\## Step 5. Calculate hyperparameters.

Tree depth warnings may be ignored when n_eff and Rhat indicate convergence.

```{r}

\# Initialize stan hyperparamater model

sm_hyper <- stan_model(file="stan_models/hyperparam.stan", model_name='hyp',verbose=FALSE)

data_hyper <- list(K=K, mugamma1=cbind(mu1, gamma1),

mugamma2=cbind(mu2, gamma2),

n1=B, n2=B, Xmu=covariates[,-1],

Xgamma=covariates[,-1])

set.seed(2)

fit_hyper <- sampling(sm_hyper, data=data_hyper, iter=4000,

chains=4,

control=list(adapt_delta=0.85, max_treedepth=15),

cores = getOption("mc.cores", ncores))

print(fit_hyper)

post_hyper <- get_posterior_mean(fit_hyper)[,5]

```

\## Step 6. Calculate mixture distribution.

```{r}

\# Initialize stan mixture model

sm_betamixinteg <- stan_model(file="stan_models/mixture_prior_integration.stan", model_name='regpaper',verbose=F)

param1r <- NULL

param2r <- NULL

for (k in 1:K){

param1r <- rbind(param1r, c(post_hyper[1] +

sum(covariates[k,-1]*post_hyper[5:(5+B-1)]),

post_hyper[3] +

sum(covariates[k,-1]*post_hyper[(5+B):(5+B+B-1)])

))

param2r <- rbind( param2r, c(post_hyper[2] +

sum(covariates[k,-1]*post_hyper[5:(5+B-1)]),

post_hyper[4] +

sum(covariates[k,-1]*post_hyper[(5+B):(5+B+B-1)])))

}

Sigma <- matrix(c(post_hyper[5+B+B+1]^2,

prod(post_hyper[(5+B+B):(5+B+B+2)]),

prod(post_hyper[(5+B+B):(5+B+B+2)]),

post_hyper[5+B+B+2]^2), ncol=2, byrow = T )

NSAMP=500

set.seed(99)

gen_random <- rmvnorm(NSAMP, sigma=Sigma)

\# Empty data

y1 <- array(0, dim=c(0))

y2 <- array(0, dim=c(0))

N1 <- 0

N2 <- 0

w <- rep(1/K, K)

data_p = list(K=K, muphi1=param1r, muphi2=param2r, N1=length(y1),

N2=length(y2), y1=y1, y2=y2, w=w,

epsilons=gen_random, NSAMP=NSAMP)

fit_reg <- sampling(sm_betamixinteg, data=data_p ,iter=2000, chains=3,

cores = getOption("mc.cores", ncores))

print(fit_reg)

```

The mean values are comparable to the mean of mu1 (0.080) and mu2 (0.103). Next, we will examine graphical views of the joint distribution, as well as the original elicited distributions.

\## Step 7. Output aggregated p1 and p2.

```{r}

traceplot(fit_reg, pars=c("p1", "p2"))

mcmc_dens(fit_reg, pars=c("p1", "p2"))

```

\## Step 8. Output risk difference.

```{r}

samples1 <- c(fit_reg@sim$samples[[1]]$p1, fit_reg@sim$samples[[2]]$p1, fit_reg@sim$samples[[3]]$p1)

samples2 <- c(fit_reg@sim$samples[[1]]$p2, fit_reg@sim$samples[[2]]$p2, fit_reg@sim$samples[[3]]$p2)

plot(density(samples2-samples1), main="")

plot(density(sample(fit_reg@sim$samples[[1]]$p1, replace = FALSE)-fit_reg@sim$samples[[1]]$p2))

```

\## Step 9. Compare to linear pooling method

```{r}

x <- seq(0,1,length.out=100)

par(mfrow=c(1,2))

plot(x, dbeta(x, mean(alpha1), mean(beta1)), type="l", ylab="", yaxt="n")

plot(x, dbeta(x, mean(alpha2), mean(beta2)), ylab="", type="l", yaxt="n")

xvec <- seq(0,1,0.001)

curve((1/K)*dbeta(x,alpha1[1],beta1[1]) +

(1/K)*dbeta(x,alpha1[2],beta1[2]) +

(1/K)*dbeta(x,alpha1[3],beta1[3]) +

(1/K)*dbeta(x,alpha1[4],beta1[4]) +

(1/K)*dbeta(x,alpha1[5],beta1[5]) +

(1/K)*dbeta(x,alpha1[6],beta1[6]) +

(1/K)*dbeta(x,alpha1[7],beta1[7]) +

(1/K)*dbeta(x,alpha1[8],beta1[8]) +

(1/K)*dbeta(x,alpha1[9],beta1[9]) +

(1/K)*dbeta(x,alpha1[10],beta1[10]) +

(1/K)*dbeta(x,alpha1[11],beta1[11]) +

(1/K)*dbeta(x,alpha1[12],beta1[12]), from=0, to=1, ylab="Density",

xlab="p1")

curve((1/K)*dbeta(x,alpha2[1],beta2[1]) +

(1/K)*dbeta(x,alpha2[2],beta2[2]) +

(1/K)*dbeta(x,alpha2[3],beta2[3]) +

(1/K)*dbeta(x,alpha2[4],beta2[4]) +

(1/K)*dbeta(x,alpha2[5],beta2[5]) +

(1/K)*dbeta(x,alpha2[6],beta2[6]) +

(1/K)*dbeta(x,alpha2[7],beta2[7]) +

(1/K)*dbeta(x,alpha2[8],beta2[8]) +

(1/K)*dbeta(x,alpha2[9],beta2[9]) +

(1/K)*dbeta(x,alpha2[10],beta2[10]) +

(1/K)*dbeta(x,alpha2[11],beta2[11]) +

(1/K)*dbeta(x,alpha2[12],beta2[12]), from=0, to=1, ylab="Density",

xlab="p2")

```

\## Step 10. Output individual beta distributions.

```{r}

for (k in 1:K) {

png(paste0("plot", k, ".png"), width=800, height=500)

par(mfrow=c(1,2), cex=1.75)

plot(x, dbeta(x, alpha1[k], beta1[k]), type="l", ylab="Density", xlab="p1")

plot(x, dbeta(x, alpha2[k], beta2[k]), ylab="", type="l", xlab="p2")

dev.off()

}

```

\## Step 11. Joint output.

```{r}

grid_space=40

\# Helper Functions for Plotting

logit <- function(x) log(x/(1-x))

inv.logit <- function(x) 1/(1+exp(-x))

\#### function for z-axis

zprior <- function(param1, param2, Sigma, w, grid_space){

nsim=500

gen_random <- rmvnorm(nsim, sigma=Sigma)

x <- y <- seq(0,1, length.out = grid_space)

x[1] <- y[1] <- 0.005

x[grid_space] <- y[grid_space] <- 0.995

z <- matrix(rep(0, length(x)*length(x)), ncol=length(x) )

for(i in 1:length(x)){

for(j in 1:length(y)){

for(k in 1:dim(param1)[1]){

supp1 <- inv.logit(param1[k,1] + gen_random[,1])

supp2 <- exp(param1[k,2] + gen_random[,2])

supp3 <- inv.logit(param2[k,1] + gen_random[,1])

supp4 <- exp(param2[k,2] + gen_random[,2])

prod_k <- sum( dbeta(x[i], supp1*supp2, (1-supp1)*supp2) * dbeta(y[j], supp3*supp4, (1-supp3)*supp4))/ nsim

z[i,j] <- z[i,j] + w[k]*prod_k

}

}

}

z

}

param1 <- cbind(logit(mu1), log(gamma1))

param2 <- cbind(logit(mu1), log(gamma2))

z2 <- zprior(param1, param2, Sigma, w=rep(1/17,17), grid_space=grid_space)

\# 3D Joint plot

par(mfrow = c(1,3))

x <- y <- seq(0,1, length.out = grid_space)

x <- y <- seq(0,1, length.out = grid_space)

x[1] <- y[1] <- 0.005

persp(x, y, z2, theta= -60, phi=15, col = "gray79", ticktype = "detailed", nticks = 6, xlab=" ", ylab=" ", zlab=" ", cex.axis=1 )

persp(x, y, z2, theta= 0, phi=0, col = "gray79", ticktype = "detailed", nticks = 6, xlab=" ", ylab=" ", zlab=" ", cex.axis=1)

persp(x, y, z2, theta= 90, phi=0, col = "gray79", ticktype = "detailed", nticks = 6, xlab=" ", ylab=" ", zlab=" ", cex.axis=1 )

```

Output

```{r}

mcmc_dens(fit_reg, pars=c("p1", "p2"))

plot(density(fit_reg@sim$samples[[1]]$p1), main="Overall Distribution",

yaxt="n", ylab="", xlim=c(0,1), xlab="Rehospitalization Probability")

lines(density(fit_reg@sim$samples[[1]]$p2), col="red")

legend(legend=c('0.60 mg/kg', '0.15 mg/kg'), col=c('black', 'red'), lty=c(1,1), "topright")

```

R Markdown file ends:

Stan file (hyperparam.stan) begins:

// This code comes from Thall et al. (2017)

data {

int<lower=1> K; // number of expert

real mugamma1[K,2]; // matrix of mu and gamma, each row is one expert, first arm

real mugamma2[K,2]; // matrix of mu and gamma, each row is one expert, second arm

int<lower=1> n1; // number of covaraite for mu

int<lower=1> n2; // number of covaraite for phi

matrix[K,n1] Xmu; // matmu

matrix[K,n2] Xgamma; // matmu

//real<lower=0,upper=1> w[K]; // weight

}

transformed data {

matrix[K,2] mugamma1transf;

matrix[K,2] mugamma2transf;

for (k in 1:K){

mugamma1transf[k,1] = log(mugamma1[k,1]/(1 - mugamma1[k,1]));

mugamma2transf[k,1] = log(mugamma2[k,1]/(1 - mugamma2[k,1]));

mugamma1transf[k,2] = log(mugamma1[k,2]);

mugamma2transf[k,2] = log(mugamma2[k,2]);

}

}

parameters {

real upsilonMU[2]; // one for each arm

real upsilonGAMMA[2]; // one for each arm

vector[n1] betaMU; // parameters for mu

vector[n2] betaGAMMA; // parameters for gamma

real<lower=-1,upper=1> rho; // correlation

vector<lower=0.001>[2] tau;

matrix[K,2] Epsilon; // randome effects for expert

real<lower=0.001> sigma0;

}

model {

vector[2] meankj1;

vector[2] meankj2;

matrix[2,2] Sigma;

Sigma[1,1] = tau[1]^2;

Sigma[2,2] = tau[2]^2;

Sigma[2,1] = tau[1]*tau[2]*rho;

Sigma[1,2] = tau[1]*tau[2]*rho;

for (k in 1:K){

meankj1[1] = upsilonMU[1] + row(Xmu,k)*betaMU + Epsilon[k,1];

meankj1[2] = upsilonGAMMA[1] + row(Xgamma,k)*betaGAMMA + Epsilon[k,2];

row(mugamma1transf,k) ~ multi_normal(meankj1,diag_matrix(rep_vector(sigma0,2)));

meankj2[1] = upsilonMU[2] + row(Xmu,k)*betaMU + Epsilon[k,1];

meankj2[2] = upsilonGAMMA[2] + row(Xgamma,k)*betaGAMMA + Epsilon[k,2];

row(mugamma2transf,k) ~ multi_normal(meankj2,diag_matrix(rep_vector(sigma0,2)));

}

// priors

upsilonMU ~ normal(0,100);

upsilonGAMMA ~ normal(0,100);

betaMU ~ normal(0,10);

betaGAMMA ~ normal(0,10);

rho ~ uniform(-1,1);

tau ~ inv_gamma(1,1);

for (k in 1:K) {

row(Epsilon,k) ~ multi_normal(rep_vector(0,2), Sigma );

}

}

Stan file ends:

Stan file (mixture_prior_integration.stan) begins:

// This code comes from Thall et al. (2017)

data {

int<lower=0> N1; // number of previous patients in first arm

int<lower=0> N2; // in the second arm

array[N1] int y1; // binary vector for fist arm

array[N2] int y2;// for the second arm

int K; // number of expert

real muphi1[K,2]; // matrix of mu and phi to be integrated, each row is one expert, first arm

real muphi2[K,2]; // matrix of mu and phi to be integrated, each row is one expert, second arm

array[K] real<lower=0,upper=1> w; // weight

//cov_matrix[K] Sigma; number random effect expert

int<lower=0> NSAMP;

array[NSAMP,2] real epsilons;

}

parameters {

real<lower=0.001,upper=0.999> p1; // prob of success first arm

real<lower=0.001,upper=0.999> p2; // prob of success second arm

}

transformed parameters {

real Delta;

Delta = p2 - p1;

}

model {

vector[K] mixture;

real supp[4];

// Likelihood

y1 ~ bernoulli(p1);

y2 ~ bernoulli(p2);

// Prior

for (k in 1:K){

mixture[k] = 0;

for (i in 1:NSAMP) {

supp[1] = inv_logit(muphi1[k, 1] + epsilons[i, 1]);

supp[2] = exp(muphi1[k, 2] + epsilons[i, 2]);

supp[3] = inv_logit(muphi2[k, 1] + epsilons[i, 1]);

supp[4] = exp(muphi2[k, 2] + epsilons[i, 2]);

mixture[k] += exp(beta_lpdf(p1 | supp[1] * supp[2], (1 - supp[1]) * supp[2]))

* exp(beta_lpdf(p2 | supp[3] * supp[4], (1 - supp[3]) * supp[4]));

}

mixture[k] *= w[k] / NSAMP;

}

target += log(sum(mixture));//modified

}

Stan file ends:
